# Self-Assembled Monolayers in Inverted Perovskite Solar Cells: A Rising Star with Challenges

**DOI:** 10.1007/s40820-026-02089-9

**Published:** 2026-02-09

**Authors:** Lele Li, Jiaqi Shi, Huimin Xiang, Xunchang Wang

**Affiliations:** Key Laboratory of Flexible Optoelectronic Materials and Technology of the Ministry of Education, Jianghan University, Wuhan, 430056 Hubei People’s Republic of China

**Keywords:** Inverted perovskite solar cells, Self-assembled monolayers, Molecular design, Stability, Large-scalable fabrication

## Abstract

Recently, self-assembled monolayers (SAMs) have been confirmed as a promising hole-selective contact and interfacial modifier for inverted perovskite solar cells (IPSCs), contributing to an inspiring record power conversion efficiency close to 27%, along with excellent stability. This review demonstrates the critical role of SAMs in enhancing the performance of IPSCs. First, the structure–property and structure–stability relationship of SAMs is systematically expounded by examining their electronic structure, spatial configuration, and the resulting intermolecular forces. Second, it concludes the underlying mechanisms how their unique properties promote the performance of IPSCs, including energy-level alignment, defect passivation, improved interface carrier extraction/transport, and the suppression of ion migration. Third, the applications of SAMs in IPSCs are systematically summarized, covering their roles as hole-selective contacts, interface modifiers, and as components in Co-SAMs strategies. Large-scalable fabrication methods are also summarized to promote industrial processing of IPSCs. Finally, the prevailing challenges and future research directions are outlined, proposing a roadmap for designing SAM-based IPSCs with superior longevity. By critically evaluating the pivotal role of SAMs, this review provides a strategic framework to guide future research and accelerate the development of self-assembled molecules in high-performance and stable photovoltaic devices.

## Highlights

- Structure–property relationship of self-assembled monolayers (SAMs) is thoroughly elucidated, including chain length, anchoring groups, linker groups, terminal functional groups, and packing density—and their resulting physical and electrochemical properties (e.g., wettability, adhesion, and electronic characteristics).
- The mechanism of SAMs on promoting the performance of inverted perovskite solar cells is discussed from the perspective of energy-level alignment, defect passivation, carrier transfer dynamics, and inhibition of ion migration.
- Perspectives and challenges of SAMs are proposed, highlighting promising directions in developing new in situ characterizations, advanced molecular designs, and optimized deposition strategies for large-scalable fabrication.

## Graphical abstract

**Figure:**
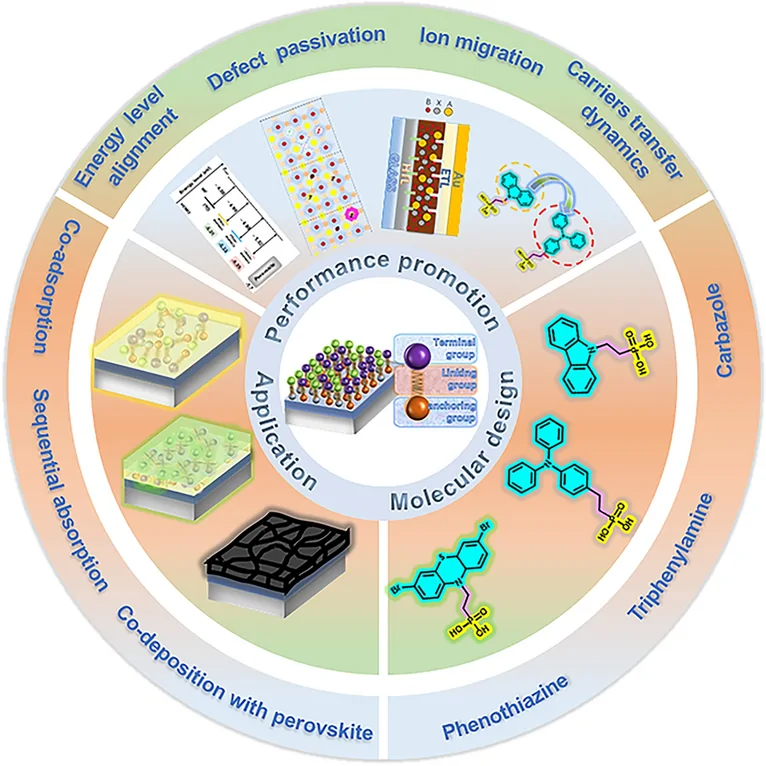

## Introduction

Inverted perovskite solar cells (IPSCs) have experienced prosperous development since the employment of self-assembled monolayers (SAMs), with a power conversion efficiency (PCE) close to 27% [1]. SAMs are formed by the spontaneous adsorption of organic molecules onto a solid substrate, resulting in a highly ordered film that is typically one molecule thick. The thermodynamic favorability of the process ensures that the molecules adopt a primarily vertical orientation on the surface. The application of SAMs in IPSCs experienced a notable surge in research and development activity after groundbreaking studies emerged in 2018 [2, 3]. In that study, a phosphonic acid (-PO(OH)₂)-based SAM was employed as a hole-selective contact (HSC) in perovskite photovoltaics for the first time. Afterward, numerous SAMs featuring carbazole-derived functional groups, specifically (2-(9H-carbazol-9-yl)ethyl)phosphonic acid (2PACz) and [2-(3,6-dimethoxy-9H-carbazol-9-yl)ethyl]phosphonic acid (MeO-2PACz) were reported [4–7]. Devices based on these SAMs outperformed those using conventional polymer-based HTLs, such as PTAA [1, 8]. Recently, studies have demonstrated that SAMs can serve as an effective alternative to conventional nickel oxide (NiO_x_) HSCs, successfully avoiding Ni^3^⁺ triggered redox and deprotonation reactions with iodide-based hybrid perovskites [9–11]. The diversity of SAM molecules enables the customized optimization of interfacial physical and photochemical properties in perovskite photovoltaics through precise molecular structure design, thereby simultaneously enhancing device efficiency, stability, and reproducibility. In addition, ultrathin monolayer of SAMs offers conformal coating, low optical and resistive losses, and versatility in interface modification.

**Fig. 1:**
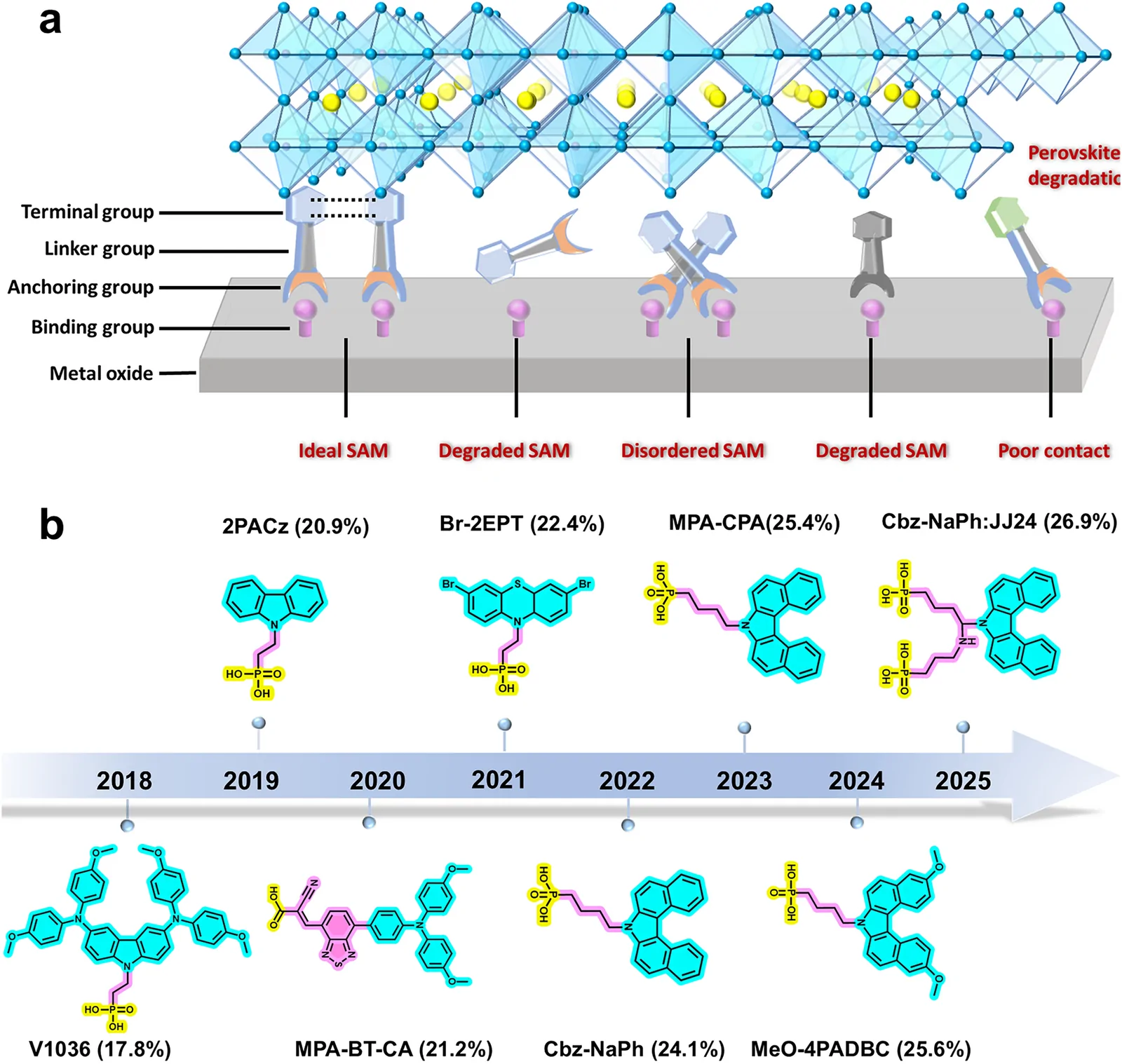
**a** Schematic diagram of the service failure in SAM-based buried interface. **b** Development and evolution of SAMs-based IPSCs [1, 2, 23]

While the monolayer architecture of SAMs undoubtedly enables near-ideal interfacial contact with minimal parasitic losses, this structural simplicity imposes inherent vulnerabilities [12, 13]. As shown in Fig. 1a, the ultrathin nature of SAMs makes interfacial properties critically dependent on molecular integrity, where even minor degradation or desorption events can trigger catastrophic failure at the perovskite/metal oxide (PVK/MO) interface. At PVK/MO interface, SAMs may desorb, degrade, and arrange in disorder under external stress. In addition, the chemical bonding between SAM and MO substrate may be disrupted under external stimulus. Such degradation pathways often lead to direct contact between adjacent functional layers, resulting in escalated leakage currents, accelerated non-radiative recombination, and other performance-degrading phenomena [14, 15]. Furthermore, the minimalist molecular design and low molecular mass of typical SAM compounds inherently limit their robustness against operational stress. This is particularly consequential in inverted photovoltaic architectures, where incident photons primarily pass through the SAM layer before reaching the perovskite absorber. This configuration subjects SAM molecules to intensified photothermal stress, potentially exceeding their stability thresholds under realistic operating conditions [16, 17]. Besides, it is difficult to cover the substrate completely owing to complex intermolecular and molecule–substrate interactions, such as limited binding sites, steric effects, and aggregation [18]. Recently, numerous studies have been conducted to develop novel SAMs molecular structure, new deposition methods to overcome aforementioned issues [19–21]. For example, Hu et al. [22] developed guanidine-modified polyurethane siloxane elastomer, interacting with 2-(3,6-dimethoxy-9H-carbazol-9-yl)ethyl]phosphonic acid (MeO-2PACz) and co-constructing a self-assembled composite structure (SACS), which enhanced anchoring ability of SAMs on substrate. This SACS boosted PCE of devices up to 26.37%, accompanied with negligible decline in performance after storage for at least 5000 h. Jen et al. [1] developed Co-SAMs methods that perfectly improved molecular packing behavior, achieving a champion PCE of 26.92%. Figure 1b illustrates evolution of SAM-based IPSCs, showing the increase in PCE from 2018 to 2025. The number of investigations into SAMs has risen significantly. Thus, it is urgent to summarize cutting-edge design strategies and guide the development of new materials [8, 23–26].

In this review, we present chemical structures of SAMs recently utilized in IPSCs and clarify their structure–properties relationship. We demonstrate the mechanism of SAMs on promoting efficiency and stability of IPSCs from following aspects: energy-level matching, bulk and interface defects passivation, carrier transfer dynamics, and inhibition of ion migration. Additionally, we summarize the applications of SAM molecules in IPSCs. Finally, we discuss the design principle of SAMs and address current challenges of SAMs, including sensitivity of SAMs-based IPSCs, charge recombination induced by deterioration of interfacial contact, and other deleterious effects and provide an outlook for the future. This review is expected to offer in-depth insights into the structure–performance correlation of SAMs, thus accelerating the rational design of novel SAM materials and promoting their commercial implementation in highly efficient and stable IPSCs.

## Fundamental Understanding Self-Assembled Monolayers

### Structure–Property Relationship of SAMs

SAM molecules consist of three basic components: an anchoring group, a linking group, and a terminal group (Fig. 1a). The anchoring group is typically a Brønsted–Lowry acid, such as a carboxylic acid (CA), a boric acid (BA), a sulfonic acid (SA), or a phosphonic acid (PA). It binds to the surface of substrate through chemical reactions and coordination interactions [27]. The linker group, usually consisting of an alkyl chain or an aromatic ring, connects the terminal group to the anchoring group, which provides flexibility/solubility and freedom for the formation of an optimally assembled structure with a stable conformation [28]. The terminal groups determine the unique characteristics and functionalities of SAMs, such as morphology, surface energy, and surface chemistry [29]. The intermolecular interactions (π–π interactions, van der Waals forces, dipole–dipole interactions, etc.) among individual SAM molecules govern the packing properties such as tilt angle and surface lattice reconstruction. The inherent flexibility of SAM molecules enables dynamic structural behaviors, including conformational isomerism, lateral diffusion, and stimulus-responsive reconstruction in reaction to changes in the environment.

#### Anchoring Groups

Anchoring groups form chemical bonds or non-covalent bonds through physical adsorption with the substrate to ensure the stable attachment. Depending on the type of substrate used for SAM deposition, anchoring groups capable of forming chemical bonds can be classified into two main categories: those designed for metals and those suited for oxides. Currently, widely used anchoring groups on oxide substrates include PA, CA, and SA.

The type and number of anchoring groups significantly influence the bonding strength. To systematically investigate this relationship, researchers designed a series of phenothiazine-based SAMs featuring identical linker and terminal groups but varying anchoring groups [27]. Theoretical calculations revealed that the PA anchoring group exhibits the lowest adsorption energy compared to SA, CA-based SAMs. This lower energy barrier facilitates faster adsorption kinetics and higher molecular loading capacity, thereby promoting the formation of a densely packed monolayer with improved surface coverage and structural integrity. As a result, IPSCs fabricated with PA-based SAM showed the highest PCE (21.43%) and the best stability among counterparts. To date, PA has been a ubiquitous anchoring group for SAM formation. A notable drawback, however, is its corrosive effect on ITO substrates, which can erode the surface and induce indium dissolution, adversely affecting conductivity [30]. Therefore, a new triphenylamine-based SAM—(4-(di-ptolylamino)phenyl)boronic acid (MTPA-BA) was developed, featuring a boronic acid anchoring group. This novel molecule exhibits a superior adsorption energy compared to both SA and CA, despite possessing lower Brønsted–Lowry acidity. Due to reduced acidity, MTPA-BA treated ITO samples exhibited minimal changes in sheet resistance compared to those modified with 2PACz. This improvement ultimately resulted in a fivefold increase in device storage stability, achieving a T₉₀ shelf life over 2400 h [31].

**Fig. 2:**
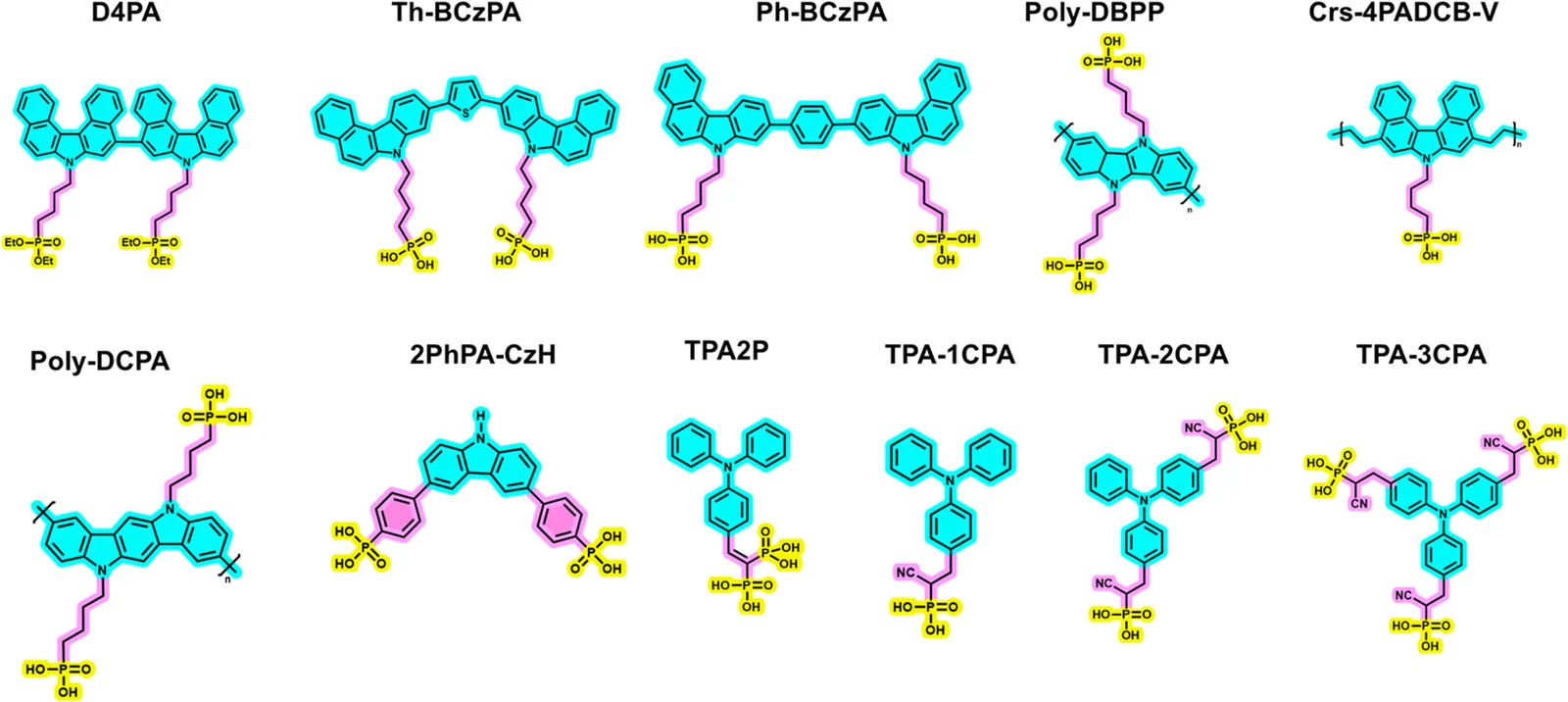
SAMs with multiple anchor groups recently reported

In general, anchoring strength increases with the number of anchoring groups. For example, a series of triphenylamine derivatives functionalized with single, double, and triple cyanoethyl phosphonic acid (CPA) groups were synthesized, designated as TPA-1CPA, TPA-2CPA, and TPA-3CPA, respectively. They were combined with the widely adopted Me-4PACz, aiming to achieve highly efficient and stable devices. TPA-3CPA: Me-4PACz-based SAM composite realized dual-sided anchoring at perovskite/SAM/substrate interface due to TPA-3CPA disrupting the aggregation behavior of Me-4PACz. As a result, TPA-3CPA-based device yielded a PCE of 26.27% with good light stability [32]. GAO et al. [33] developed a bidentate-anchored superwetting aromatic SAM, utilizing a carbazole core, designated as 2PhPA-CzH ((9H-carbazole-3,6-diyl)bis(4,1-phenylene))bis(phosphonic acid). Theoretical calculations revealed 2PhPA-CzH reacted with the ITO substrate via two P-O-In bonds. Devices based on 2PhPA-CzH retained above 90% of their initial PCE after 1000 h in ambient air (∼85%RH, 85 °C), compared with 80% retention for MeO-PhPACz counterparts. Dimeric poly-SAMs and poly-SAMs were reported to anchor strongly on substrate due to multiple repeated anchoring units, which will be discussed in the following sections [34–38]. The recently reported SAMs structures with multianchor groups are shown in Fig. 2. Attaching multiple phosphonic acid anchoring groups onto a triazatruxene core can form three PA groups in a tripodal based SAM, strongly bonding to the ITO substrate in a bidentate attachment mode [39]. With the P=O group uncoordinated to the surface, the presence of three anchoring groups induced a face-on orientation of the molecules, resulting in the formation of a densely packed and effective monolayer. The rational design of SAMs structures not only enhanced the anchoring capabilities but also promoted the formation of highly ordered molecular arrangements on the substrate surface [40, 41].

The bonding between SAM molecule and substrate relies critically on the affinity of their anchoring groups for hydroxyl sites on metal oxide surfaces. Therefore, establishing a sufficient density of stable anchor sites is essential to ensure robust SAM—substrate interaction. 2PACz and Me-4PACz tend to form unstable bonds on TCO surfaces, which is attributed to the intrinsically low density of anchoring sites on these substrates [42]. Moreover, even when formed, bonds at weak sites are susceptible to desorption by strong polar solvents [43]. The hydroxylation pretreatment of TCO substrates is typically performed by sequential sonication in deionized water, acetone, and isopropanol, followed by an ultraviolet (UV) ozone or plasma treatment for several minutes, with hydroxylation occurring in the last step [44, 45]. Zhao et al. [46] employed hydroxylation etching to increase the hydroxyl anchor sites on ITO, which was achieved by soaking the substrate in a mixed solution of H_2_SO_4_ and H_2_O_2_. Hydroxylated ITO constructed a more efficient, stable anchoring strength with MeO-2PACz, enabling the fabrication of PSCs with a PCE of 26.6%. The resulting devices retained 98% of their initial PCE after 1000 h of damp-heat testing (85 °C, 85% RH, following the ISOS-D-3 protocol) and maintained 93% of the initial PCE after 1600 h of thermal cycling between room temperature and 85 °C. Fang et al. [47] demonstrated immersing FTO substrates in H_2_O_2_ solutions with UV illumination could increase –OH density. UV illumination activated H_2_O_2_ molecules to generate reactive –OH radicals, which then energetically bonded with the FTO surface, constructing a hydroxyl-rich interface for homogeneous deposition and adsorption of SAMs. The –OH seeding strategy effectively homogenized the deposition of SAMs on the substrate, leading to superior buried interface contact and high‑quality perovskite films. Benefiting from these improvements, the resulting PSCs achieved a champion PCE of 26.19%. The devices also demonstrated significantly enhanced operational stability, retaining nearly the same efficiency with a loss of less than 7% after 1000 h of continuous MPPT (under ISOS-L-3 protocol at 65 °C) using encapsulated target devices. You et al. [48] improved the dispersion of NiO_x_ nanoparticles and increased their hydroxyl anchor site density by treating them with H_2_O_2_. As a result, the subsequently developed NiO_x_/SAM bilayer HSCs exhibited robust bonding capability. By inserting an ultrathin ALD-NiO_x_ buffer layer between ITO and the spin-coated NiO_x_ film, the surface hydroxylation of the latter was significantly enhanced. This led to a dramatic increase in surface hydroxyl groups providing abundant high-quality anchoring sites and thereby facilitating the formation of superior SAM films [49].

#### Linking Groups

Linking groups between the hole-selective unit and the anchoring group act as a bridge connecting the anchoring groups and terminal groups. Linking groups can be categorized into alkyl chains and π-conjugated systems. While a long insulating alkyl linker tends to hinder hole transfer from the perovskite layer to the conductive substrate, a short alkyl chain enhances hole extraction efficiency but fails to effectively block electrons, resulting in increased charge recombination. In 2019, Albrecht et al. [8] synthesized 2PACz with a brief carbon chain (*n* = 2). 2PACz with short-chain length showed rapid hole extraction, while the overly truncated chain also caused disordered molecular packing, which compromised its interfacial passivation performance. Afterward, the same group systematically compared the charge transfer kinetics of n-PACz series with varying alkyl chain lengths (*n* = 2, 4, 6). 2PACZ exhibited a significantly higher hole transport rate than 6PACz due to its shorter tunneling distance, while the long-chain structure enhanced molecular ordering. Therefore, the design of alkyl chain length needs to consider the trade-off between charge transport and interfacial passivation, with C2–C4 being the optimal range. It should be noted that excessively elongated chains can adversely affect electrical conductivity [50]. 4PACz possesses both efficient charge transfer and ordered packing, which may account for the generally superior performance over 2PACz with its shorter ethyl linker in PSCs. It also offers a plausible explanation for the scarcity of reports involving alkyl chain linkers longer than n-butyl. Generally, short-chain SAMs exhibit good solubility in polar solvents (e.g., tetrahydrofuran, isopropanol, and chloroform), while long-chain SAMs dissolve more readily in nonpolar solvents (e.g., toluene and n-hexane). Mixed solutions can be employed for further optimization. Additionally, the odd–even effect in the linker groups alter molecular packing, thereby modifying the electronic coupling [51, 52]. The resulting structure, physical, and chemical properties of SAMs vary depending on the parity (i.e., whether n is odd or even) of the number n of methylene (–CH_2_–) units constituting the alkyl chain and present periodic oscillation phenomenon. SAMs with even-numbered chains are typically more tilted relative to the substrate normal than those with odd-numbered chains [52]. Even-numbered chain SAMs typically exhibit higher interchain order and fewer conformational defects. This is because the molecular tilt and rotational conformations adopted by even-numbered chains enable more stable and tighter intermolecular van der Waals interactions within the hexagonal close-packed lattice. In contrast, the arrangement of odd-numbered chains is relatively more prone to some disorder [51]. This observed phenomenon may account for the empirical finding that odd-numbered alkyl chains are rarely employed as HSC, as their structural properties could be less ideal for this specific function.

In contrast, π-conjugated linkers exhibit strong charge delocalization effects. This makes them a superior choice for SAMs, as they enable efficient carrier extraction and resulting in a fully conjugated SAM structure, which not only shows enhanced carrier mobility but also improved long-term stability under 1-sun illumination at elevated temperatures conditions [24, 53]. The aromatic spacers were among the first types employed in SAM designs due to their auxiliary aromaticity, which can enhance the properties of the functional terminal group [54, 55]. Xu and co-workers developed a novel SAMs—[4-(3,6-dimethyl-9H-carbazol-9-yl)phenyl]PA (Me-PhpPACz) by replacing flexible butyl linker with a rigid conjugated phenylene ring in Me-4PACz [56]. Due to the rigid structure of the phenylene ring, Me-PhpPACz molecules arrange more tightly on the substrate, forming a denser film that effectively reduces leakage current and enhances stability of IPSCs. The π-conjugated phenylene ring improved the efficiency of intermolecular charge transport, increasing the FF and thereby boosting the device PCE from 24.14% to 26.17%, accompanied by excellent stability. Similarly, Gao et al. [28] employed new self-assemble molecule, MeO-2PACz, which featured a conjugated linker in place of the ethyl linker in MeO-2PACz. MeO-PhPACz showed larger dipole moment (2.89 D) than MeO-2PACz (1.31 D), which enhanced hole extraction and transport efficiency. The optimal device based on MeO-PhPACz and wide-bandgap perovskite achieved a PCE of 21.10%, higher than the MeO-2PACz counterparts (19.53%). More importantly, the inherent UV resistance and stronger UV absorption capacity of the MeO-PhPACz structure enable the target IPSCs to exhibit superior UV stability compared to control devices, which is crucial for large-scale outdoor operations. Overall, π-conjugated linker groups overcome the insulating property and flexibility of alkyl linkers, which not only enhances charge transport but also stabilizes the electron-rich arylamines through electron/charge delocalization [54].

**Fig. 3:**
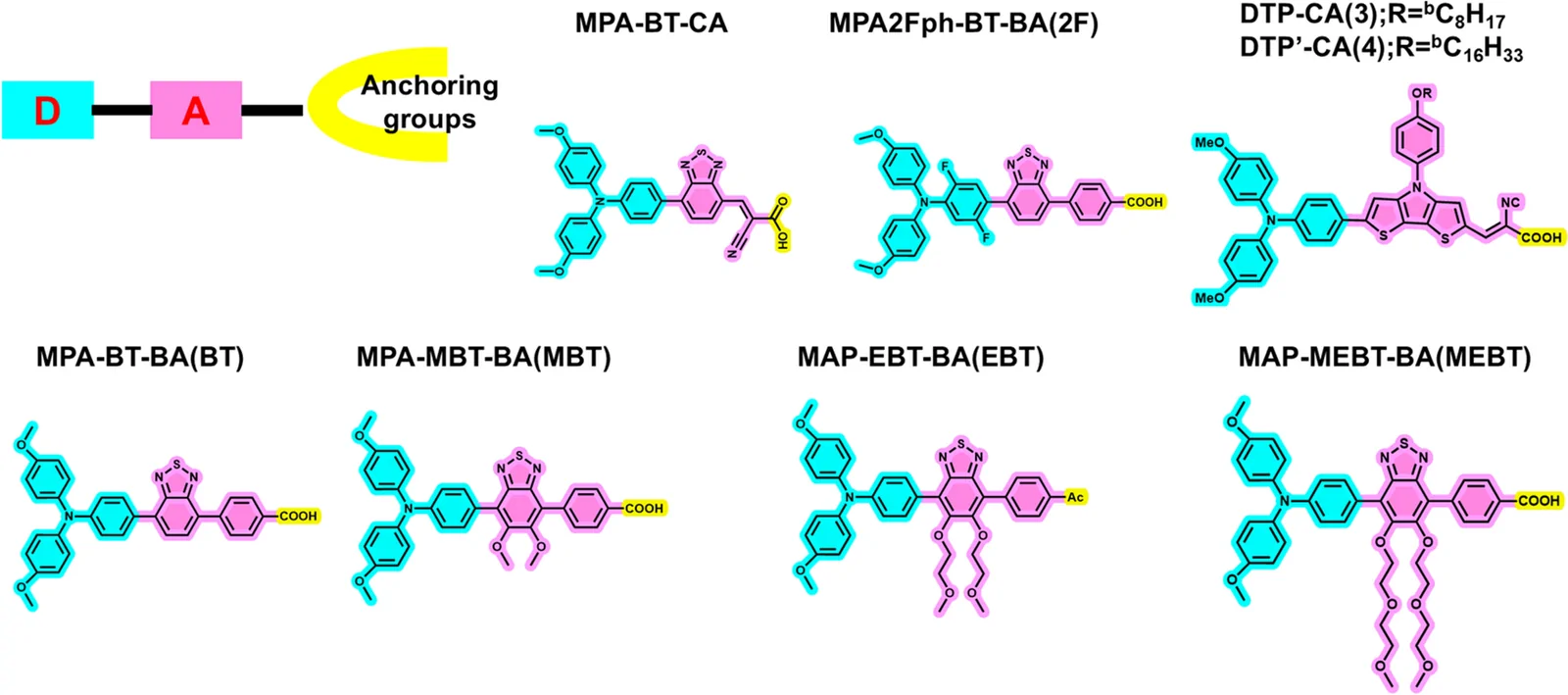
D-A type linker-based SAMs recently reported

Inspired by the structural design of dye molecules in dye-sensitized solar cells, Wang et al. [26] designed a donor (D)-acceptor (A) type of SAMs, (3-(7-(4-(bis(4-methoxyphenyl)amino)phenyl)benzo [*c*][1,2,5]thiadiazol-4-yl)-2-cyanoacrylic acid), simplified as MPA-BT-CA. MPA serves as an effective donor with good oxidative ability and high hole-transporting efficiency. The acceptor benzothiadiazole (BT) acts as linker groups, featuring a planar structure that enhanced intermolecular stacking and sulfur atoms of BT is capable of interacting with undercoordinated Pb^2^⁺ ions via coordination bonds. To date, most of SAMs are suitable for pure-Pb devices. Developing SAMs with adjustable dipole moment, molecular stacking mode and suitable energy level is critically important for tandem photovoltaic devices. Building on this design, the same group developed D-A type SAMs 4-(7-(4-(bis(4-methoxyphenyl)amino)-2,5-difluorophenyl)benzo[c][1,2,5]thiadiazol-4-yl) benzoic acid, named MPA2FPh-BT-BA, featuring BT as linker groups and two F atoms on donor unit. MPA2FPh-BT-BA aimed to address interface issues of wide-bandgap (WBG) and low-bandgap (LBG) perovskites. In LBG Sn–Pb devices, the introduction of F atoms decelerated crystallization and, by virtue of their stronger and more selective coordination with Sn^2^⁺ than Pb^2^⁺ ions, effectively suppressed Sn^2^⁺ oxidation, thereby improving the perovskite film quality. In WBG-based devices, 2F effectively passivated the cation defects at the 2F/WBG interface and inhibited non-radiative recombination [57]. Afterward, they introduced a series of oligoether side chains with different lengths (methoxy, 2-methoxyethoxy, and 2-(2-methoxyethoxy)ethoxy) to the BT unit. This modification ultimately produced the desired D-A type SAMs, abbreviated as MBT, EBT, and MEBT and designed for LBG perovskite-based IPSCs. EBT exhibited a face-on dominant orientation on ITO, accompanied by a higher dipole moment, which facilitated efficient charge extraction, modulated crystal growth, and passivated surface defects in Sn–Pb perovskite films. These combined effects contributed to improved device performance [58]. The chemical structure of recently reported D-A type SAMs is shown in Fig. 3.

#### Terminal Groups

Typical SAMs with a carbazole terminal group possess intrinsic properties, including molecular dipoles, energy levels, and packing order, that play a crucial role in governing the perovskite film quality [59]. The lone pair electrons on carbazole terminal group can coordinate strongly with uncoordinated Pb^2^⁺, passivating trap states and reducing non-radiative recombination. The carbazole group constitutes an electron-rich, large π-conjugated system with high overall electron density, which allows for the construction of weak electrostatic interactions with uncoordinated I⁻ ions potentially through hydrogen atoms on the benzene rings. This weak electrostatic interaction further contributes to the stabilization of the crystal structure.

Terminal groups design can be divided into three categories according to their structures and functions: (i) Electron donor/hole transfer types such as carbazole group [8], triphenylamine groups (–TPA) [11], and phenothiazine and its derivatives groups [60]. A characteristic of these terminal groups is their π-electron-rich aromatic amine structure, which consists of electron-donating nitrogen atoms and a large, three-dimensional molecular framework. The huge structure can inhibit excessive aggregation between molecules, forming high-quality films without pinholes. (ii) Defect passivated terminal groups (–CH_3_, –NH_2_, −SH, –O–, etc.). Simple inert end groups, such as −CH_3_, can physically shield the underlying sensitive substrate or active layer by forming a dense, hydrophobic molecular barrier that blocks environmental water and oxygen, thereby preventing degradation and defect generation. Chemical passivation is a more direct and effective approach, where the terminal groups interact with the defect sites on the surface via Lewis acid–base interaction or coordination bonds. Lewis base end groups (e.g., −NH_2_,  –SH, –O–) can act as electron donors and coordinate with positively charged, uncoordinated Lewis acid sites on the surface (e.g., Pb^2^⁺, Sn^2+^) to fill their empty orbitals and thereby “neutralize” the defect. Conversely, Lewis acid end groups can passivate surface negative charge defects. (iii) Energy-level modulation and interfacial dipole functional terminal groups (halogen, etc.) [61, 62]. The diversity of available terminal groups enables precise fine-tuning of the surface work function (WF). Specifically, electron-donating groups (e.g.,–NH_2_, –CH_3_) lower the WF, whereas electron-withdrawing groups (e.g., –CF_3_, –CN) increase it, thereby facilitating the construction of an energy-level gradient. Terminal groups of SAMs directly contact and interact with the overlying active layers. Therefore, selecting an appropriate head group is critical for modifying the interface properties and ultimately enhancing device performance. Through organic synthesis, a series of functional groups in SAMs can be introduced to exchange with specific atoms (O, N, S, I, Br, and Cl) in perovskite layers. Moreover, these interactions could modify the surface work function and tailor the energy levels, thereby enhancing the charge extraction and suppressing interfacial carrier recombination. It is well-established that intermolecular interactions are highly vulnerable to thermal stress, resulting in disorganized molecular packing. This disorder could diminish dipole alignment, reduce hole mobility and undermine the interfacial integrity between the metal oxide and perovskite layers. Thus, it is essential to carefully elaborate the linker and terminal group to balance the contradictory requirements of processability and structural order.

### Structure–Stability Relationship of SAMs

#### Thermal Stability

As mentioned above, SAMs are more easily degraded than inorganic HSCs under external stress such as UV light and thermal stress. The thermal stability of SAM molecules is dictated by their binding strength to the substrate, as thermal energy drives the dissociation of bonds linking the anchoring groups and spacers. For a specific SAMs, the thermal stability is not solely determined by the anchor bond strength, but also ultimately depending on the most vulnerable point within its molecular framework. In a comparison of SAMs with –S, –Se, and –COO anchors, Cyganik et al. [63] found that naphthylthiols exhibited the highest thermal stability. With a desorption energy of 1.69 eV, its stability not only ranked the highest among the groups studied but also surpassed that of most reported N-heterocyclic carbene (NHC) monolayers, which are widely regarded as the most stable SAMs on metal substrates. Increasing anchoring sites has been demonstrated to improve thermal stability of SAMs. For example, Ge et al. [37] designed a dimeric SAM named Ph-BCzPA, consisting of the monomer BCzPA((4-(7H-benzo[c]carbazol-7-yl)butyl) phosphonic acid) to improve anchoring strength on substrate. Ph-BczPA with two PA anchoring groups showed robust anchor capability on substrate. The corresponding device achieved a PCE of 26.33%, maintaining 90% of its initial value at 85 °C with a RH of 60%–70% after 2000 h aging. SAM molecule with conjugated triphenylamine linkers showed excellent thermal and morphological stability. Their small molecular motion is beneficial for achieving a high glass transition temperature, thus mitigating their degradation under thermal stress [64]. Poly-SAMs also exhibit good stability due to their repeat unit with multiple anchoring and terminal groups, which will be discussed in Sect. 5.

#### UV Stability

UV light can destroy weak chemical bonding between perovskites and hole-transporting materials, leading to poor interfacial charge transfer and accelerated ion migration [65, 66]. To enhance the bonding strength, Huang et al. [67] designed an aromatic phosphonic acid, [2-(9-ethyl-9H-carbazol-3-yl)ethyl]phosphonic acid (EtCz3EPA). The PA groups were linked to the –OH groups on ITO substrate, whereas –NH–, =O, halide (–X), and carbazole groups interacted with Pb^2+^in the perovskites. This multisites chemical bonding enhanced efficiency and improved the UV stability of IPSCs. UV exposure has been found to trigger the functional degradation of SAMs, characterized by a direct deterioration in their hole extraction ability. This effect originates from UV-induced bond rupture near the carbazole terminal, which undermines the structural integrity of monolayer and thereby its electronic function [28]. Besides, light-induced phase segregation of WBG perovskite has been widely noticed and systematically studied [68]. By employing phenyl groups as linking units, π-conjugated SAMs can enhance carrier transport properties while also significantly boosting their intrinsic UV resistance [28, 56]. For example, Yang et al. [69] developed a thiophene modified π-conjugated SAMs named Me-TPCP, which features phenyl linking groups instead of the conventional alkyl linking groups. This molecular design enhanced UV stability and hole transport efficiency by extending the overall π-conjugation of the SAM, shielding the carbazole portion from degradation, and providing a more conductive molecular framework. As a result, pristine and Me-TPCP-based devices retained approximately 51.3% and 80.4% of their initial PCEs after 72 h under prolonged UV exposure, respectively.

### Aggregation Behavior of SAMs

#### Self-Assembly Process

Before discussing molecular stacking and aggregation behavior, we will first expound on the formation process of SAMs. The self-assembly process of SAM molecules generally occurs in two stages: initial adsorption and subsequent rearrangement and reorientation. In the initial stage, SAM molecules rapidly and efficiently anchor to the substrate surface through covalent bonding. Following this, the SAM molecules undergo rearrangement and reorientation to minimize the system’s free energy, ultimately forming a highly ordered monolayer [70]. The intrinsic properties of the SAM molecules and environmental factors (such as temperature, humidity, and solvent polarity) are critical to the SAMs assembly process [71].

SAM molecules exhibit inherent amphiphilicity, and their self-assembly mechanism resembles that of conventional surfactants [72]. For surfactants, monolayer formation typically requires the concentration to remain below the critical micelle concentration (CMC). Above the CMC, molecules preferentially aggregate into micellar structures, thereby impeding uniform monolayer deposition [73]. Enlarged aromatic hydrophobic moieties such as carbazole groups generally promote stronger intermolecular interactions compared to alkyl chains, yielding a higher CMC and enhanced structural stability [74]. However, enhanced van der Waals forces and π–π interactions of aromatic hydrophobic units collectively contribute to the formation of incomplete island-like domains [18].

An optimal solution temperature enhances molecular diffusion and adsorption kinetics, while humidity and solvent polarity collectively modulate interactions between the molecules, the substrate, and the surrounding environment, thereby affecting the assembly rate and the quality of the resulting film. Interfacial wettability is further governed by the chemical structure of the SAM molecules and the surface characteristics of the substrate. Suboptimal wettability occurs when the polarity of the SAM molecules does not align with that of the substrate surface, or when the molecules lack functional groups with sufficient hydrophilic/hydrophobic to effectively mediate interactions with the solvent and substrate. This poor wettability leads to non-uniform spreading of the SAM solution, resulting in inhomogeneous film thickness and the formation of defects including pores and cracks.

#### Origin of Aggregation Behavior

Aggregation behavior is the result of the competition and collaboration of various forces on a two-dimensional plane. These forces contain chemical bonding between anchoring groups and substrate, van der Waals forces, and other interaction between adjacent molecules, as well as molecule-solvent interactions. At conventional processing concentrations, SAM molecules often assemble into micellar nanoparticles in solution rather than remaining as individually dispersed molecules. These micelles are prone to mutual aggregation or even precipitation from solution, which significantly compromises the shelf stability of SAM inks for practical use [18]. Introducing polar cosolvents or coordinating additives can improve the solubility of SAM molecules and alter molecule-solvent interactions, preventing aggregation [38, 75].

Traditional film fabrications such as spin coating depend on fast solvent evaporation and centrifugal force. In this process, the chemical bonding between anchoring groups and hydroxy groups enables rapid substrate attachment, while the spin-coating speed and time can be precisely adjusted to control film thickness and uniformity. Weak bonding of SAMs—substrate may lead to intermolecular aggregation rather than a perpendicular arrangement on the substrate. The planar and π-conjugated nature of many SAM molecules, such as carbazole and TPA derivatives, often leads to strong π–π stacking in solution, causing aggregation prior to deposition. As more molecules accumulate, weak repulsive forces between them help form aggregations and clusters. Intermolecular aggregation stems from π–π stacking interactions as well as the specific contributions of terminal groups of adjacent molecules [76]. Tuning the intermolecular interactions to mitigate undesired self-aggregation can be accomplished through several strategies: introducing steric hindrance groups, optimizing side-chain length, designing an asymmetrical structure, or incorporating specific substituents designed to attenuate excessive intermolecular interactions [7, 76–79].

Conjugated linker groups enhance hole mobility via tight π–π stacking but often contributes to less compact packing. Alkyl chains facilitate dense packing while hindering charge transport. Simultaneously achieving high hole transport efficiency and compact packing order remains an ongoing challenge in the field. Strong π–π interaction leads to aggregates (such as micelles or π-association complexes) in solution. These aggregates form uneven “island-like” or “domain-like” structures during film formation process, which disrupts the continuity of the film. Besides, under influence of thermodynamics, these aggregates undergo further structural rearrangement and energy optimization, resulting in crystal domain regions with long-range order and periodic lattice structure. Hou et al. [80] systematically investigated the influence of crystalline and amorphous SAMs on perovskite growth, interfacial charge transfer, and the performance of IPSCs. They compared two types of SAMs: Me-4PACz (crystalline, c-SAM) and amorphous PhPACz (a-SAMs), the latter featuring a rotatory phenyl group. Their study demonstrated that the amorphous phase of SAMs enables more uniform perovskite growth. Amorphous SAMs development can be realized via augmenting steric hindrance and weakening intermolecular interactions between molecules. It is acknowledged that crystalline SAMs, by providing ordered yet discrete nucleation sites, can result in uneven grain sizes and grain boundary aggregation within the perovskite layer. Nevertheless, their well-ordered molecular structure facilitates efficient vertical charge transport. This explains why many high-performance photovoltaic devices have been achieved using SAMs containing a substantial proportion of crystalline domains. In contrast, a-SAMs provide a uniform and continuous nucleation interface that promotes homogeneous nucleation and vertical growth of perovskite, resulting in larger and more consistent grains. Although the disordered structure avoids the grain boundary barriers, providing more uniform lateral and longitudinal charge extraction, the absolute charge mobility may be slightly lower than c-SAMs.

## How Self-Assembled Monolayers Promote IPSCs Performance

SAMs have emerged as a powerful and versatile strategy for enhancing the performance and stability of IPSCs. They are typically employed at charge-transporting interfaces (e.g., between the perovskite film and electrode, HSC). The mechanism of action is multifaceted and can be divided into four key areas: (1) energy-level alignment and optimization, (2) defect passivation, (3) carrier transfer dynamics, and (4) inhibition of ion migration. To systematically clarify the mechanism of SAMs on performane of IPSCs, we first disscuss the characterazation methods for the composition, orientation, thickness, coverage  rate, distribution of functional groups  and interface properties of SAMs.

### Methods for Characterizing Self-Assembled Monolayers

The quality of the SAMs, including molecular orientation, surface morphology, packing, and substrate anchor bonding, directly determines device performance. However, due to their unique monolayer structure, conventional characterization methods such as optical and morphological techniques are generally less effective for SAMs than for bulk materials. Accurately characterizing SAM film quality and obtaining microscopic details remain challenging, impeding both device fabrication and practical application. Inadequate characterization further risks poor reproducibility, especially for researchers new to the field, due to a lack of clear procedural guidance. To address these limitations, this section reviews mainstream characterization techniques for SAMs in solution-processed thin-film electronics. These techniques mainly cover four categories according to their mechanisms: (1) surface analysis/interaction, (2) electron microscopy, (3) photoelectron spectroscopy, and (4) optical spectroscopy.

#### Surface Interaction

**Fig. 4:**
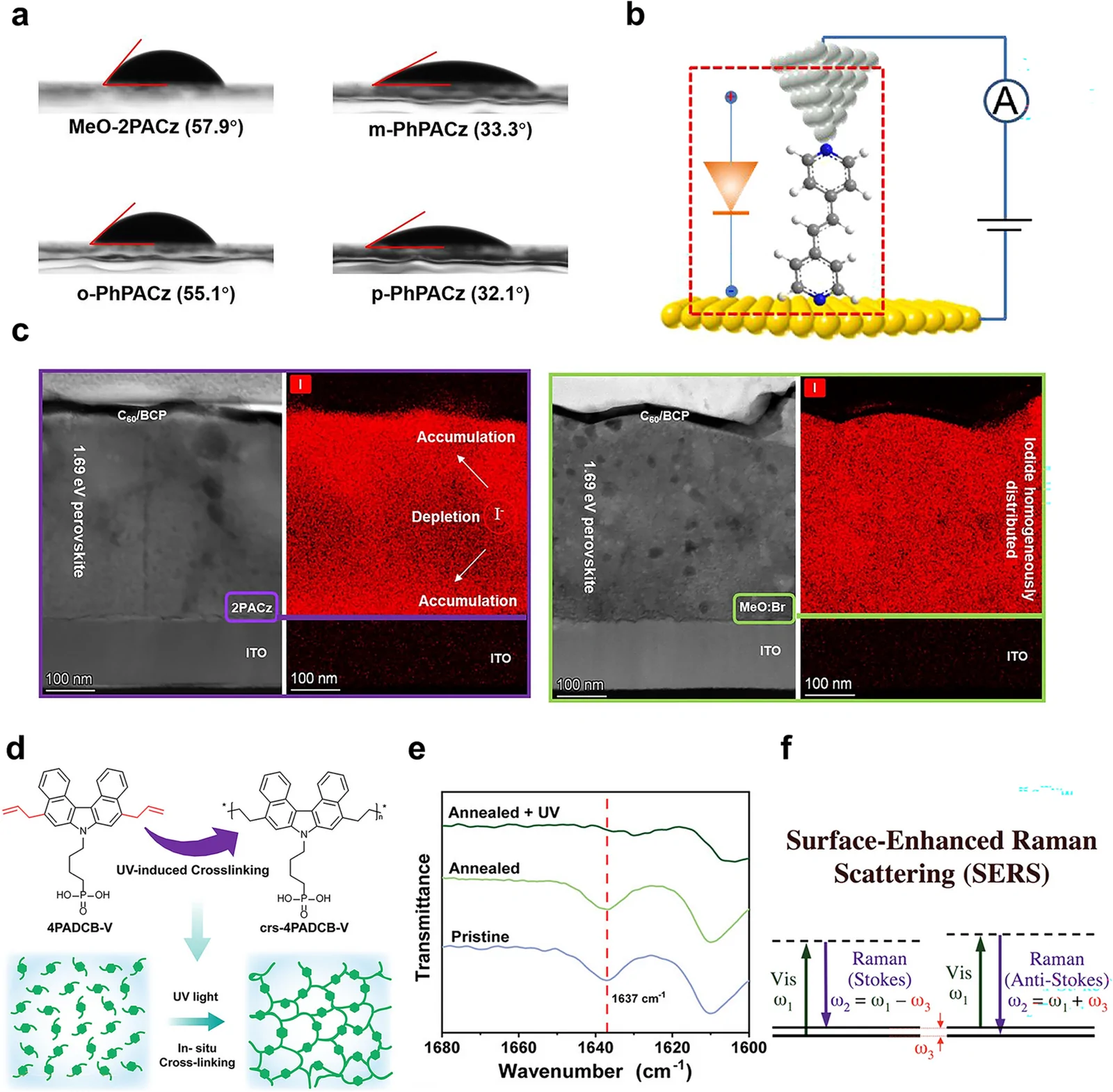
**a** Perovskite precursor solutions contact angles images of different SAMs on FTO. Reproduced with permission [81]. Copyright 2025, American Chemical Society. **b** Working mechanism of STM. Reproduced with permission [83]. Copyright 2021, American Chemical Society.** c** Cross-sectional HAADF-STEM image with corresponding STEM-EDS of complete device stack after 40 min of MPPT. Reproduced with permission [88]. Copyright 2021, Elsevier Ltd. **d** Schematic illustration of UV-induced cross-linking. Reproduced with permission [91]. Copyright 2025, Wiley–VCH. **e** FTIR spectra of 4PADCB-V before and after thermal annealing and UV irradiation.** f** Schematic diagram of SERS due to molecular vibrations. Reproduced with permission [93]. Copyright 2012, American Chemical Society

Conventional surface interaction testing includes contact angle measurements, scanning probe microscopy (SPM). Contact angle measurements reflect the substrate-surface wettability based on Youngʼs equation and describe the interactions and surface energy relationships among the liquid, solid, and vapor phases. Water or other liquids are used as liquid phase. The wettability of SAMs can be directly correlated to the contact angle values, which are related to the hydrophilicity of the terminal groups, linker groups, and molecular packing density. The contact angles of the water droplets on MeO-2PACz, o-PhPACz, p-PhPACz, and m-PhPACz were measured as 57.9°, 55.1°, 32.1°, and 33.3°, respectively. The lower contact angles of m-PhPACz indicated its superior wettability and uniform growth on the FTO surface, which was beneficial for achieving uniform and complete coverage of the perovskite (Fig. 4a) [8]. In thiophene oligomer-based SAMs, the water contact angle showed a positive correlation with chain length. Longer alkyl chains effectively shield the substrate by minimizing water access to unbonded sites, indicating that SAMs with a higher degree of polymerization tend to form denser, more complete monolayers [82].

SPM refers to a family of microscopy techniques where a sharp physical probe is mechanically raster-scanned across a sample surface with subnanometer precision, achieved through piezoelectric actuation. As a widely used approach for surface characterization, SPM encompasses various methods. Scanning tunneling microscopy (STM) and atomic force microscopy (AFM) are widely used methods to probe electronic structures and surface morphology of SAMs. In STM, a bias voltage is applied between the tip and the sample surface, and the resulting tunneling current is measured as a function of tip position. This allows STM to map the electronic structure of the interface at a specific energy level (Fig. 4b) [83]. It should be noted, however, that STM reveals electronic structures rather than actual atomic surface morphology. Currently, STM has been widely used in studying the mechanism of SAMs on solid surfaces, including assembly conformation, solvent selection, and phase transition [34].

Similar to STM, AFM operates primarily in two modes: constant force mode and constant height mode. To minimize artifacts and distortions caused by cantilever positioning offsets in the x–y plane, especially under fluctuating temperature, AFM measurements are typically conducted under ambient atmospheric conditions and at room temperature. Crystalline organic monolayers, which exhibit well-defined shapes and boundaries, are typically fabricated from conjugated molecules and characterized by AFM. A step-like height profile can be directly observed, revealing a height difference approximately equal to the length of SAM molecule [57]. AFM can also be employed to characterize SAMs by analyzing their surface topography [35, 38, 85]. For example, AFM observations revealed that spin-coated oligothiophene SAMs exhibited a small number of aggregates on the substrate surface. These aggregates could be effectively eliminated by solvent rinsing or alternative post-processing methods [86]. Observing step-like height profiles in organic monolayer crystalline films remains challenging, particularly when the functional head group of the SAM has a small volume or weak conjugation. Therefore, complementary techniques such as contact angle measurement are often required for comprehensive SAM characterization. With a conductive tip, conductive AFM can detect the current in contact mode to assess SAM growth [21, 81].

#### Electron Microscopy

The most widely used electron microscopy techniques are scanning electron microscopy (SEM) and transmission electron microscopy (TEM). Due to their high energy, which is detrimental to organic materials, electron beams are not ideal for direct SAM characterization. However, electron microscopy can be applied under specific conditions. For example, when combined with an energy-dispersive X-ray spectroscope (EDS), it can characterize elemental species and their relative quantities [25, 87].

TEM is an imaging technique that generates contrast by transmitting an electron beam through an ultrathin specimen and recording its interaction with the sample. This method requires samples to be sectioned into very thin slices, typically with a thickness between 50 and 100 nm. Owing to the short wavelength of electrons, TEM can achieve a resolution as high as 0.2 nm. When combined with EDS, TEM can provide more comprehensive information about SAM thin films. In STEM image in Fig. 4c, an accumulation of iodide at both the HSC and ETL interfaces was observed for 2PACz-based PSCs, with an iodide depletion region at the center of the bulk perovskite. However, no obvious iodide accumulation was observed for MeO-2PACz and Br-2PACz mixed SAMs-based PSCs [88]. In high-resolution TEM (HRTEM) image, SAM is clearly distinguishable from adjacent layers. The measured thickness of approximately 2.5 nm closely matches the expected thickness of a compressed assembly layer, confirming the formation of a dense and well-ordered SAM [89].

#### Photoelectron Spectroscopy

Photoelectron spectroscopy, commonly referred to as photoemission spectroscopy, involves measuring the kinetic energy of electrons ejected via the photoelectric effect to determine the binding energies of electrons within a target material. Based on the energy source employed for ionization, photoelectron spectroscopy is primarily divided into two types: ultraviolet photoelectron spectroscopy (UPS) and X-ray photoelectron spectroscopy (XPS).

UPS analyzes the kinetic energy spectra of photoelectrons emitted from molecules following ultraviolet photon absorption, enabling the determination of molecular orbital energies within the valence band region [47, 81, 87]. XPS is a sensitive method for detecting solid surfaces, which can probe up to an average depth of ~ 5 nm in an ultrahigh vacuum environment. In XPS analysis, elements that are not inherently present in the substrate are often used as references. These include phosphorus in PA anchors, metals within complex functional groups, sulfur in conjugated moieties and thiol groups, as well as halogens introduced through structural modifications [26, 36, 90]. The distinct peaks corresponding to these elements are typically well-resolved in XPS spectra, providing direct evidence for the ultrathin architecture of the SAM.

#### Optical Spectroscopy

Optical spectroscopy includes two categories: emission spectroscopy and absorption spectroscopy. Fluorescence spectroscopy, a widely used characterization technique based on emission spectroscopy, enables the analysis of SAM formation when the monolayer incorporates chromophores such as aggregation-induced emission groups, conjugated structures, or bioluminescent moieties. By monitoring variations in fluorescence intensity, lifetime, and spectral peak positions, the growth and structural evolution of such SAMs can be effectively evaluated [21, 34, 91, 92]. Typical characterizations for optical spectroscopy include (i) Fourier transformation infrared spectroscopy (FTIR) and (ii) Raman spectroscopy.

In an FTIR measurement, the sample is irradiated with a broadband infrared beam, typically covering the mid-infrared range from 400 to 4000 cm^−1^. Fourier transform of the time-dependent intensity signal converts this interferogram into a conventional spectrum of intensity versus wavelength (or wavenumber). The development of FTIR has significantly advanced infrared absorption spectroscopy. Unlike traditional dispersive spectrometers, which rely on slits and monochromators to scan wavelengths sequentially, FTIR collects spectral information across all wavelengths simultaneously. This multiplex (Fellgett) advantage, together with higher optical throughput (Jacquinot advantage), results in faster measurements, improved signal-to-noise ratio, and higher sensitivity, making FTIR particularly effective for analyzing thin organic films such as SAMs. To simplify fabrication process and protect sample surface, attenuated total reflectance (ATR) takes advantage of a spectral acquisition technique that utilizes the total internal reflection phenomenon and interacts with the sample. ATR-FTIR is commonly employed to study the assembly regularity and bonding mode of alkyl SAMs. For example, Xu et al. [91] reported the UV-induced in situ polymerization of 4PADCB-V (crs-4PADCB-V) by ATR-FTIR characterization (Fig. 4d). The C=C stretching vibration peak at 1637 cm^−1^ showed pronounced attenuation in crs-4PADCB-V films exposed to 365 nm UV light, whereas thermally treated samples exhibited spectra nearly identical to pristine 4PADCB-V (Fig. 4e).

Raman spectroscopy analyzes the light scattered by a sample to detect quantum-level energy gains or losses resulting from molecular excitations. This technique is widely employed to obtain vibrational information of materials. However, its inherently weak signal is often obscured by other stronger signals, such as elastic (Rayleigh) scattering and fluorescent, which limits its applications in interfacial studies [93, 94]. To address this, the surface-enhanced Raman spectroscopy (SERS) has been applied to characterize SAMs deposited on rough metal surfaces or metal nanoparticles, benefiting from their capability in amplifying signals (Fig. 4f). These investigations enable the identification of chemical species, structural configurations, and adsorption orientations, as well as the real‑time monitoring of anions, surfactants, environmental contaminants, biomolecules, and dye molecules during chemical or electrochemical reactions. For example, Pemberton et al. [95] examined the molecular conformation of alkylsilane SAMs by utilizing the enhanced ν(C–C) and ν(C–H) Raman signals on a SiO₂‑modified Ag substrate, which provided substantial electromagnetic enhancement. Furthermore, SERS can be integrated with STM to implement tip‑enhanced Raman spectroscopy (TERS), thereby enabling spatially resolved Raman mapping. In one notable study, Zenobi et al. [96] developed a TERS‑based hyperspectral approach to visualize the on‑surface decomposition process of a pyridine‑4‑thiol SAM on atomically flat Au(111) under ambient conditions, achieving spectroscopic detection with a spatial resolution of about 5 nm. This work established a new avenue for quantitatively investigating on‑surface decomposition chemistry and associated dynamic processes. Despite its utility, Raman spectroscopy’s inherently low signal‑to‑background ratio, especially in metal‑based SERS systems, often necessitates signal enhancement. This requirement can introduce experimental complexity and potential contamination, posing challenges for in situ device fabrication and subsequent characterization.

### Energy-Level Alignment and Optimization

Achieving efficient charge extraction is highly dependent on a suitable energy-level alignment between the charge transport and perovskite layers. Modifying the substrate with SAMs alters this alignment by inducing interfacial charge redistribution, which shifts the work function. This shift in work function (Δ*φ*) can be calculated as follows [97]:

1 $$\Delta \varphi =-N\left[\frac{{\mu }_{\perp SAM}}{{\varepsilon }_{0}{k}_{SAM}}+\frac{{\mu }_{S-M}}{{\varepsilon }_{0}{k}_{S-M}}\right]$$

where *N* is the adsorbate grafting density,${\mu }_{\perp SAM}$ is the vertical component of the molecular dipole moment, *ε₀* is the vacuum permittivity, and *k* is the dielectric constant of the adsorbate layer. Since the term ${\mu }_{S-M}$/(${\varepsilon }_{0}{k}_{S-M}$) is largely determined by the properties of the substrate, Δφ is primarily a function of the dipole moment of the SAM normal to the surface (${\mu }_{\perp SAM}$). By strategically engineering this interfacial dipole, the work function can be precisely tuned. This adjustment directly affects the quasi-Fermi level splitting under illumination, which is directly correlated to the open-circuit voltage (*V*_OC_). Moreover, the optimized energy alignment facilitated by the SAM accelerates charge extraction and minimizes energy losses at the interface. This enhancement in carrier dynamics simultaneously boosts the short-circuit current density (*J*_SC_) and the fill factor (FF), leading to an overall improvement in device performance.

**Fig. 5:**
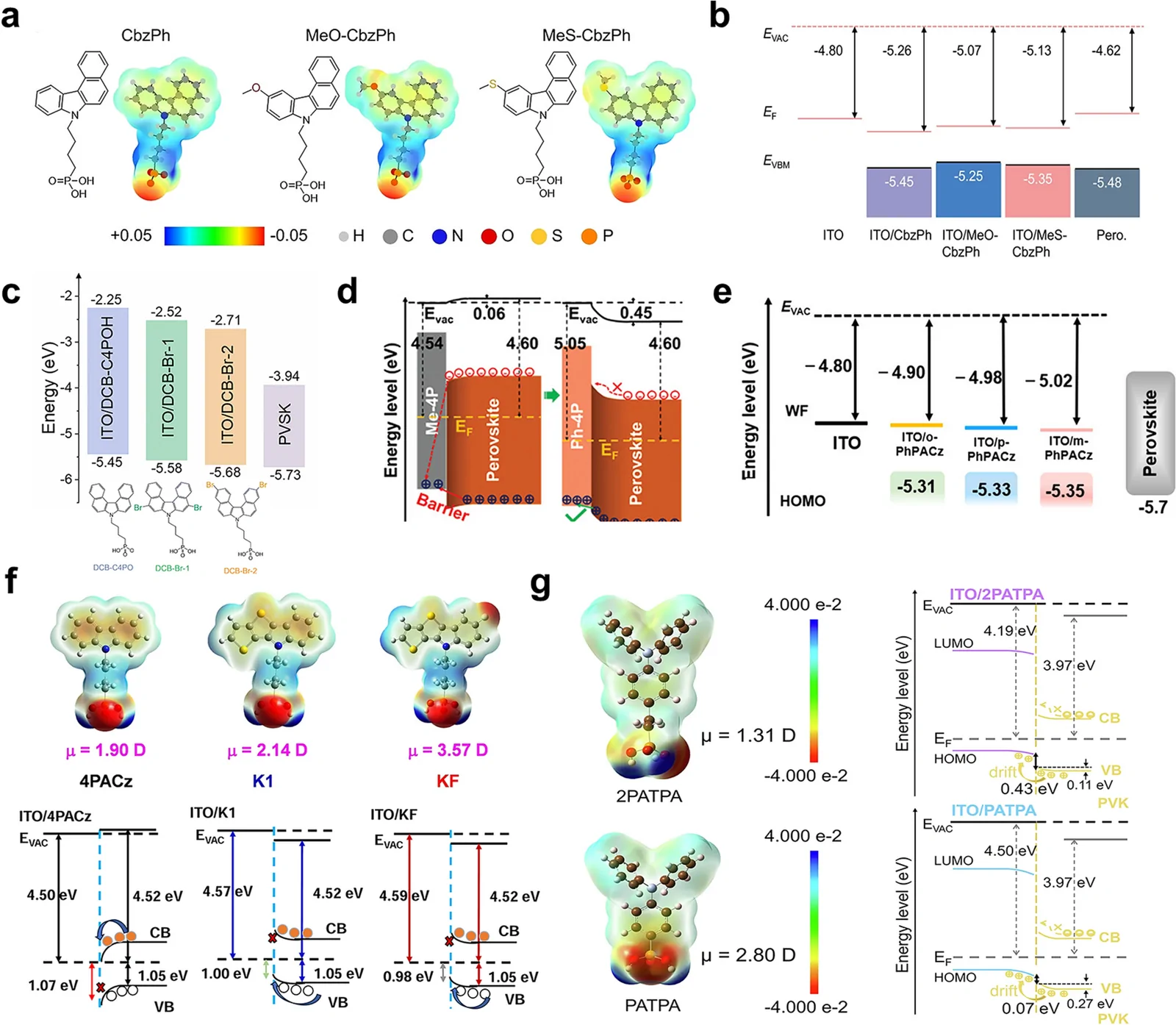
**a** Chemical structure and ESP of CbzPh, MeO–CbzPh, and MeS–CbzPh and **b** its corresponding energy levels. Reproduced with permission [100]. Copyright 2024, Wiley–VCH. **c** Energy-level diagram of different DCB-C4POH and its Br substitutes-based architectures. Reproduced with permission [102]. Copyright 2024, Royal Society of Chemistry. **d** Energy-level diagrams of Me-4PACz and Ph-4PACz-based architectures. Reproduced with permission [40]. Copyright 2023, Wiley–VCH. **e** Schematic diagram of energy-level arrangement of ortho, meta, andpara-PhPACz-based architectures. Reproduced with permission [81]. Copyright 2025, American Chemical Society. **f** Chemical structure, dipole moment of 4PACz, K1, and KF and their schematic diagram of energy-level arrangement. Reproduced with permission [103]. Copyright 2024, Wiley–VCH. **g** Chemical structure, dipole moment of PhpPACz, 2PATPA, and PATPA and their schematic diagram of energy-level arrangement. Reproduced with permission [9]. Copyright 2025, Springer Nature

WBG perovskite-based solar cells serve as essential components in tandem architectures, holding promise for surpassing the Shockley–Queisser efficiency limit. However, these devices frequently exhibit substantial *V*_OC_ losses, largely attributed to non‑radiative recombination and suboptimal interfacial energy alignment. In p-i-n structured devices, the enhanced halide segregation within wide-bandgap perovskites, driven by interfacial recombination, constitutes a primary cause for the degradation of inverted perovskite solar cell stability [98, 99]. Interfacial properties are tightly related to light stability of WBG perovskite-based cells. Charges generally transfer to their respective electrodes. However, holes tend to accumulate within the perovskite at the HTL/perovskite due to an energy barrier at valence band maximum (VBMs) between the SAM and perovskite interface. This accumulation leads to an electric field that pulls more anions toward the HSC interface compare to the case of aligned VBMs. Similarly, in the presence of an energy‑level offset, a high density of electrons accumulates within the perovskite layer at the HSC/perovskite interface, which in turn draws cations toward this interface. Charge accumulation impacts halide segregation, and ultimately despairs long-term photostability of PSCs [88]. Laquai et al. [88] proposed MeO-2PACz and Br-2PACz mixed SAMs exhibited faster hole transfer rates, which could tune energy matching at the HTL/perovskite interface to increase the device performance and the device photostability in WBG PSCs. The electron-donating capacity of terminal group strongly influences the frontier orbital energy levels of SAMs. It has been observed that a mismatch exists between the highest occupied molecular orbital (HOMO) level of MeO-based SAMs and the perovskite layer, originating from the strong electron-donating capability of the methoxy group. Introducing a methylthio (MeS-) substituent constructs a more optimal HOMO level and improves hole transport properties [100]. The electrostatic potential surfaces (ESP) of the CBzPh derivatives reveal that the MeO- and MeS-groups create a negative potential near the aromatic side, with the MeS-CbzPh analog notably possessing the largest dipole moment of 3.25 D (Fig. 5a). The weaker electron-donating ability of the MeS-group provides MeS-CbzPh with a deeper HOMO level compared to MeO-CbzPh, which is beneficial for increasing the open-circuit voltage of the device (Fig. 5b). The device with the MeS-CbzPh HTL exhibited outstanding performance, achieving a high PCE of 26.01% along with excellent stability. Br-substitution on terminal group shows the similar effect of energy-level adjustments and the substitution position dictates the strength of the interaction between the resulting SAM and the WBG perovskite layer. As shown in Fig. 5c, Br-substitution on (4-(7*H*-dibenzo[*c*,*g*]carbazol-7-yl)butyl)phosphonic acid (DCB-C4POH) can lower the HOMO level and achieve better energy-level alignment. Substituting benzene rings on carbazole unit increased dipole moment of Ph-4PACz to 2.32 D, which is larger than that of Me-4PACz (1.44 D). The Ph-4PACz induced upward band bending of 0.45 eV effectively enhances hole extraction while simultaneously suppressing electron accumulation at the interface. As a result, this SAM achieves a high *V*_OC_ of 1.20 V in IPSCs, along with a notable PCE of 25.6% [40]. Furthermore, the development of SAMs with asymmetric carbazole terminal groups and rigid phenyl linkers—such as o‑PhPACz, m‑PhPACz, and p‑PhPACz, which featured methoxy‑substituted benzene rings at different positions—has a significant influence on interfacial energy level. The dipole moment and HOMO level of series SAMs are summarized in Fig. 5d–g [9, 81, 100–103].

### Defect Passivation

**Fig. 6:**
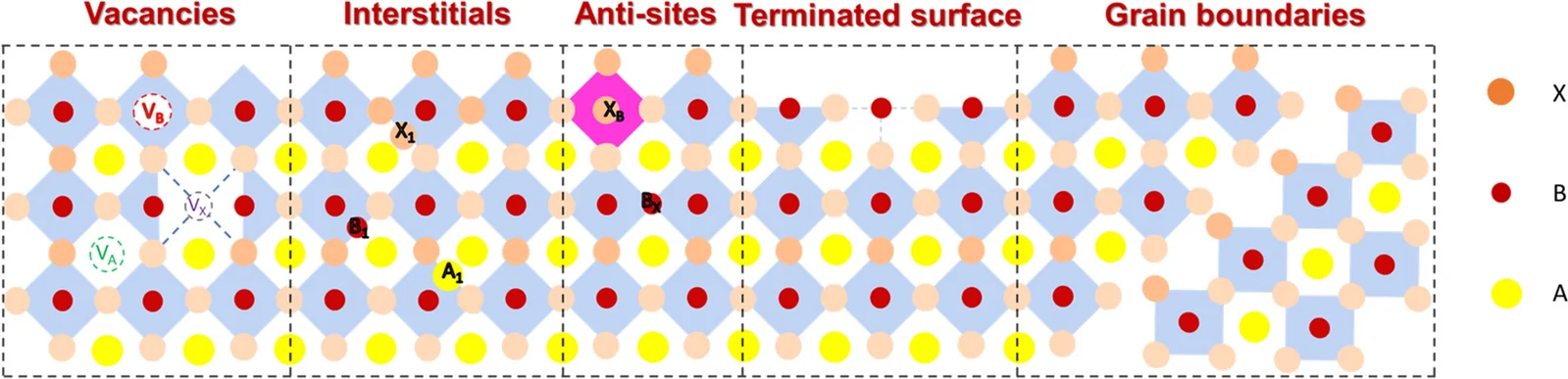
Various type of perovskite defects

Interfacial defects in perovskite/substrate or perovskite upper layer can significantly reduce the overall performance of PSCs. Defects can be classified into two types according to their dimensions and locations: point defects and surface defects. Point defects include those originating from intrinsic defects of perovskite or those introduced by foreign impurity atoms or ions (Fig. 6) [104]. For example, ABX_3_ crystals contain three types of vacancies (V_A_, V_B_, and V_X_), three interstitials (Ai, Bi, and Xi), two cation substitutions (A_B_ and B_A_), and four off-site substitutions (A_X_, B_X_, X_A_, and X_B_) [105]. Surface defects include grain boundaries, dangling bonds and interface defects from poor interface contact, energy-level mismatch, or mutual diffusion, featuring a discontinuous crystal lattice with undercoordination of the exposed ions. Defects lead to a series of severe outcomes such as hysteresis and halide phase segregation. Moreover, surface defects act as pathways for water and oxygen penetration, significantly impairing stability and efficiency of PSCs [106].

**Fig. 7:**
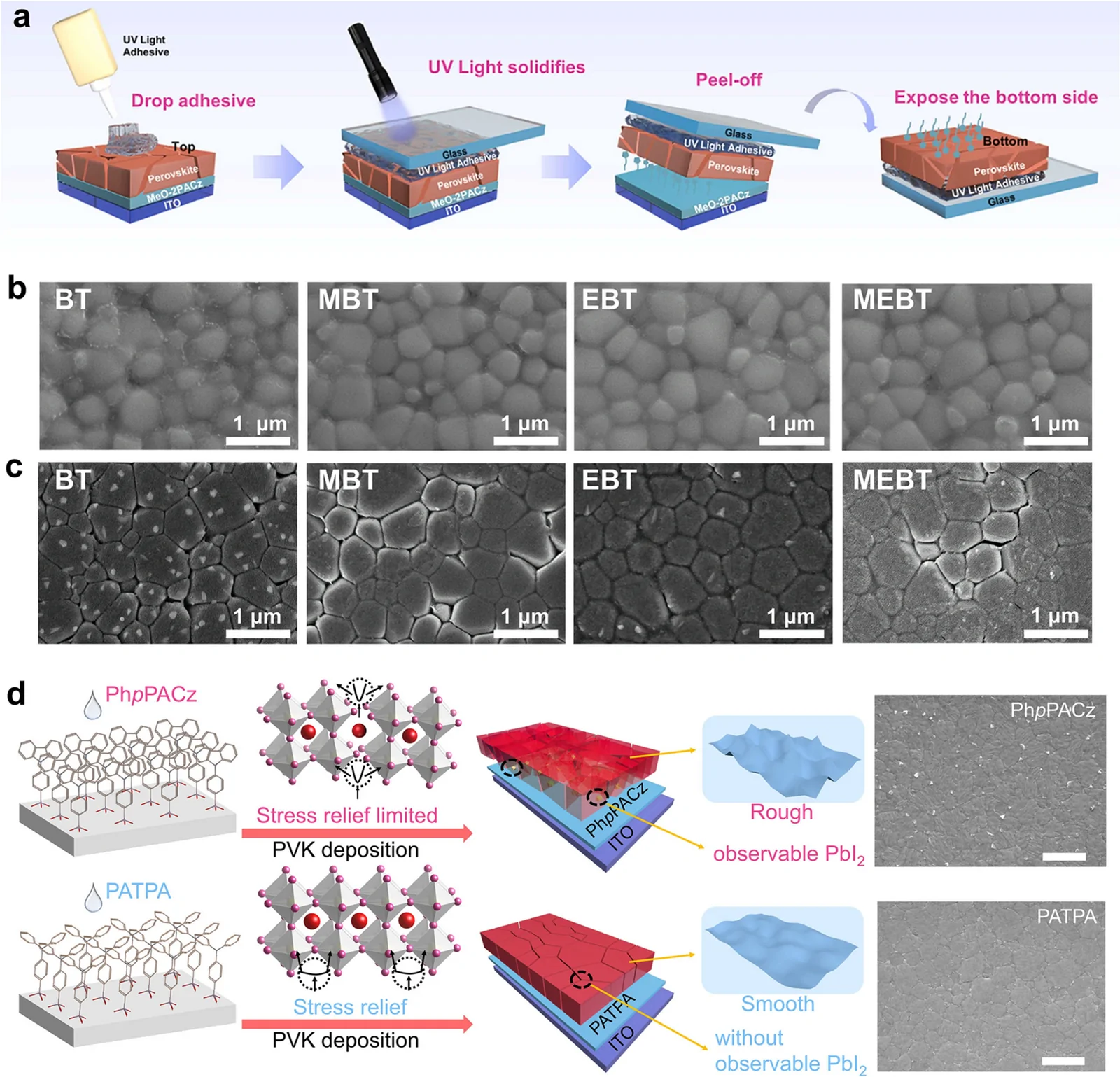
**a** Schematic diagram of the perovskite film peel off method. Reproduced with permission [108]. Copyright 2024, Elsevier Ltd. **b** Top-view and** c** buried interfaces SEM images of Sn–Pb perovskite films deposited on different HSCs. Reproduced with permission [58]. Copyright 2025, Springer Nature. **d** Schematic diagram of morphology and defect passivation of perovskite films after deposition PhpPACz and PATPA, respectively. Reproduced with permission [9]. Copyright 2025, Springer Nature

Defect passivation can be realized via rational design of anchoring and terminal groups, which interact with substrate and absorber layer, respectively. SAMs with diverse terminal groups containing heteroatoms such as nitrogen, oxygen, or sulfur can interact with uncoordinated Pb^2+^ through their lone electron pair. For example, Hong et al. [25] designed a novel phenothiazine-derived SAMs, designated as Br-2EPT ((2-(3,7-dibromo-10H-phenothiazin-10-yl)ethyl)phosphonic acid), which featured dual anchoring capabilities through Pb–S and halogen bonding with the perovskite layer. These interactions effectively suppressed interfacial trap states, leading to significantly enhanced device stability. Wolf et al. [107] applied a mixed solution of HBzA (4-hydroxybenzylamine) and 2PACz onto an ITO substrate, proposing that the adsorption of HBzA is facilitated by acid–base reactions and hydrogen bonding with both ITO and 2PACz. During the subsequent deposition of the perovskite, HBzA⁺ cations exchanged with formamidinium (FA⁺) or cesium (Cs⁺), releasing ligands that promote in situ formation of a 2D perovskite layer beneath the 3D film. This resulting 2D/3D heterostructure effectively passivated interfacial defects and suppresses ion migration, leading to markedly improved device stability under continuous light soaking and thermal stress. Lin et al. [108] reported a multifunctional organic salt, 2-(methylthio)-4,5-dihydro-1H-imidazolium iodide (MTIm), as an interfacial modifier for PSCs. As shown in Fig. 7a, peeling off perovskite films is a critical step for characterizing the buried interface. MTIm chemically reacted with excess PbI₂ to form a 1D perovskite structure, (MTIm)PbI₃, which was distributed across both the top and buried interfaces. This enabled dual-interface passivation and suppresses non‑radiative recombination. Additionally, MTIm effectively relaxed lattice strain and mitigates defect‑induced adverse effects at the buried interface. Wu et al. [19] examined the bottom interfacial of perovskite growth on different SAMs substrate (PTAA, 2PACz, MPA-CPA) by SEM (MPA-CPA: (2-(4-(bis(4-methoxyphenyl)amino)phenyl)-1-cyanovinyl)phosphonic acid). An epoxy encapsulant was employed to peel off perovskite from substrate; thereby, the buried interface of perovskite layer was exposed. Numerous nanovoids were observed at the bottom surface of the perovskite film grown on PTAA, likely due to insufficient wetting caused by the hydrophobic nature of PTAA. The number of nanovoids decreased when perovskite was deposited on the 2PACz layer. Notably, perovskite deposited on the MPA‑CPA substrate formed a highly compact and homogeneous film, completely free of observable voids, which was due to superwetting underlayer of MPA-CPA. As a result, MPA-CPA-based device achieved a certified PCE of 25.4%, higher than PTAA (22.6%) and 2PACz (23.4%) counterparts.

Oligoether side chains-based SAMs (BT, MBT, EBT, and MEBT) with different lengths were reported to regulate crystal growth, and passivate surface defects in Sn–Pb perovskite films. As shown in Fig. 7b, the surface morphology of modified perovskite films was slightly different from that of the BT/LBG film, which was attributed to crystallization regulation of Sn–Pb perovskite induced by effective oligoether side-chain engineering. In Fig. 7c, the BT/LBG film exhibited a disordered surface morphology composed of numerous small grains. In contrast, introducing side chains into BT (EBT) resulted in a homogeneous surface with compact and uniform perovskite grains. While the MEBT/LBG film showed improved homogeneity compared to BT/LBG, its grain distribution uniformity remained suboptimal, which was attributed to the relatively disordered orientation of MEBT molecules after anchoring to the ITO substrate [58]. A flexible triphenylamine terminal group with robust phenyl bridge alleviated lattice stress and reduced PbI_2_ defects density, which in turn promoted the formation of larger grain sizes and more compact perovskite films. As shown in Fig. 7d, the schematic diagram illustrated the key morphological and defect passivation contrasts between perovskite films deposited on ITO/PhpPACz and ITO/PATPA substrates. The flexible, adaptive head groups of PATPA facilitated superior interfacial contact, leading to more uniform film growth and larger grains compared to its more rigid counterparts. The terminal group serves as the principal site of direct interaction with the perovskite. Its structural configuration and contact mode are instrumental in determining the crystalline growth of the perovskite matrix, as well as the morphology and quality of the buried interface [9].

**Fig. 8:**
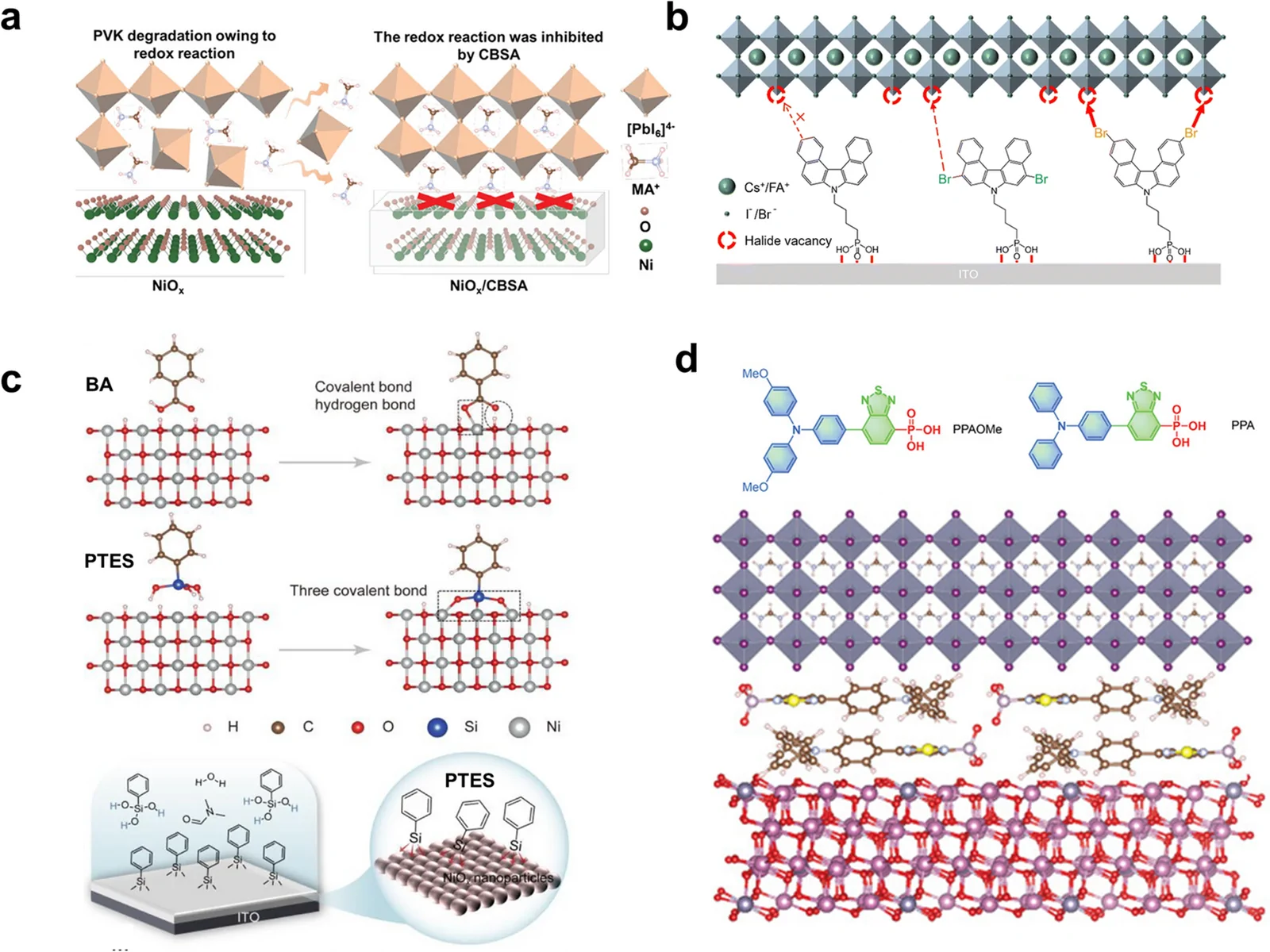
**a** Interfacial defects passivation mechanism of CBSA. Reproduced with permission [109]. Copyright 2021, Wiley–VCH. **b** Illustration of SAMs in interacting with the perovskite layer. Reproduced with permission [102]. Copyright 2024, Royal Society of Chemistry. **c** Schematic of the anchoring mechanism of BA and PTES molecules on NiO_x_. Reproduced with permission [110]. Copyright 2025, Wiley–VCH. **d** Chemical structure of PPAOMe, PPA and the zoom-in visualizes the *D–A* SAHTMs attached at the ITO/perovskite interface. Reproduced with permission [114]. Copyright 2022, Wiley–VCH

In IPSCs employing NiO_x_ as the HSC, interfacial treatment with SAMs can diminish inevitable surface defects and reduce hydroxyl groups. Furthermore, it establishes an energetically aligned interface, reducing energy loss during the hole transfer process and regulating perovskite crystallization to elevate film quality. Chen’s group introduced a SAM composed of p-chlorobenzenesulfonic acid (CBSA) to modify NiO_x_ HSCs [109]. The sulfonic acid groups of CBSA passivated oxygen vacancies on the NiO_x_ surface, while chlorine atoms filled iodine vacancies in the underlying perovskite, thereby reducing interfacial stress and suppressing detrimental reactions between NiO_x_ and perovskite (Fig. 8a). As a result, CBSA-treated devices achieved a remarkable PCE of 21.8%, along with enhanced stability under various operational conditions. Hou et al. [102] showed that the bromine in DCB-Br-2 donated a pair of non-bonding electrons to uncoordinated Pb^2^⁺ ions or halide vacancies, which enhanced the interaction with the perovskite layer and suppressed interfacial non-radiative recombination, as shown in Fig. 8b. As a result, a remarkable *V*_OC_ of 1.37 V was achieved in a 1.79 eV WBG perovskite-based cell, with a voltage loss of only 0.42 V, exceeding 90% of the Shockley–Queisser *V*_OC_ limit. Yuan et al. [110] developed a novel molecule, [4-(trifluoromethyl)phenyl]triethoxysilane (3F-PTES), which formed robust and stable Si–O–Ni covalent bonds through condensation reactions, significantly enhancing the interfacial binding strength and surface coverage compared to conventional silane-based coupling agents (Fig. 8c). Consequently, the device based on 3F-PTES maintained a PCE of 26.47% with excellent operational stability. The device retained 97% of its initial efficiency after 1500 h of continuous operation under maximum power point tracking (MPPT) condition.

### Carriers Transfer Dynamics

The carrier extraction dynamics at the SAMs/perovskite interface has significant impacts on the performance of IPSCs [111, 112]. The charge extraction rate is strongly influenced by both the anchoring geometry and the intrinsic carrier mobility of the SAM molecules. Current research efforts are predominantly directed toward tailoring the π-conjugated core and the anchoring group of SAMs to achieve compact, well-oriented monolayers at the buried perovskite interface. A face-on orientation of the π-conjugated system is particularly desirable, as it promotes efficient hole extraction in devices [20, 113]. Achieving a densely packed and highly ordered monolayer is essential for optimizing interfacial charge transfer. To this end, recent studies focus on designing novel π-expanded structures such as carbazoles, triphenylamines, and triazatruxene-based derivatives with enhanced dipole moments and stronger π–π stacking interactions.

In the case of carbazole-based SAMs, Jen and colleagues reported asymmetric or helical π-expansion (CbzPh and CbzNaph) to control monolayer packing [24]. Theoretical studies revealed that the resulting molecule, CbzNaph had the largest dipole moment (2.41 D), suitable HOMO energy level (–5.39 eV) for matching well with perovskite layer, and tight π–π stacking (π–π distance = 3.34 Å). Its dipole moment was significantly higher than that of 4PACz (1.68 D). This compact packing structure promoted the formation of the densest assembly. As a result, CbzNaph-based device achieved a champion PCE of 24.1% with improved device stability. Chen et al. [20] introduced a benzene group and a carbazole group to the original carbazole unit of 4PACz to form asymmetric SAM of 4PAbCz. 4PABCz exhibited a dipole moment of 2.06 D, slightly larger than that of 4PACz (1.98 D). In addition, the out-of-plane dipole moments of 4PABCz derived a more ordered face-on packing pattern of SAM HSCs, which facilitated hole extraction on substrates.

The π-expansion design strategy has been successfully extended to triphenylamine-based SAMs. Owing to its stronger electron-donating capability, the triphenylamine unit facilitates the formation of SAMs with a greater molecular dipole. A particularly effective approach to modifying the triphenylamine core is constructing donor–acceptor molecular architectures. Li and co-workers developed a series of π-expanded self-assembled hole-transporting molecule (SAHTM) that incorporate a triphenylamine-derived donor unit (PPA), a methoxy-functionalized donor (PPAOMe), and a benzo[c][1,2,5]thiadiazole acceptor moiety to enhance hole extraction [114]. This donor–acceptor configuration yielded large dipole moments of 6.97 and 8.86 D for PPA and PPAOMe, respectively, and thus promoting the formation of a well-ordered monolayer (Fig. 8d). Theoretical simulations further suggest that PPA molecules adopted a face-on orientation when anchored onto the ITO substrate. Leveraging these advantages, PPA-based IPSCs achieved a champion PCE of 23.2%, along with a *V*_OC_ of 1.14 V and FF of 82.0%.

Other π-conjugated systems such as quinoxaline derivatives and ruthenium-based dyes have also been developed to optimize the molecular orientation and ordering of SAMs in IPSCs. These materials promoted a face-on anchoring geometry and enhanced molecular packing, which in turn facilitated efficient hole extraction and transport. As a result, such SAMs enable an optimal balance between high *V*_OC_ and high FF, achieving PCEs exceeding 23% [41].

### Inhibition of Ion Migration

In perovskite halide crystals, point defects commonly exist as charge-compensating pairs, primarily manifested as Frenkel defects and Schottky defects. At the atomic scale, Frenkel defects consist of vacancy–interstitial pairs, whereas Schottky defects comprise pairs of oppositely charged vacancies. These defects, particularly vacancy-type defects, provide essential diffusion pathways for ion migration, including halide anions (e.g., I⁻) and metal cations (e.g., MA⁺, FA⁺) under operational stressors (light, heat, electric field) [115–117]. Ion migration is a primary degradation pathway in PSCs, triggering a cascade of detrimental effects. Ion migration further accelerates the formation of defects in the perovskite layer, which in turn promotes and enhances ion migration. This vicious cycle can induce localized stoichiometric imbalance, thereby disrupting defect equilibria and facilitate the formation of deleterious defects, which ultimately accelerates the degradation of perovskite materials.

The smooth, hydrophobic nature of conventional HSCs like PTAA often leads to vertically inhomogeneous perovskite films due to non-ideal crystal growth, which contributed to a higher degree of ion migration and thus, inferior operational stability of PSCs. Compared to PTAA, the rough texture of SAMs substrate provides more nucleation sites, which is beneficial for the formation of the intended triple cation perovskite with high vertical compositional homogeneity [118]. Ion migration occurring at both the bottom and top interfaces of perovskite layers has been widely observed [119, 120]. In response, strategic molecular design of SAMs in IPSCs has been employed to suppress the diffusion of halide ions and volatile organic species from the perovskite, preventing their penetration into the underlying SAM interface and the top ETL. The intrinsic molecular dipoles of SAMs can modulate the local electric field at the interface, lower the effective Schottky barrier, and consequently suppress ion migration. For instance, Li et al. [121] developed a molecular additive, simplified as BT-T. This additive features with triphenylamine electron donor and a benzothiadiazole-containing electron acceptor with cyanoacrylic acid as the anchoring group, which was incorporated, directly into the perovskite layer. BT-T spontaneously formed SAM, establishing stable bonds with both the perovskite lattice and the underlying metal oxide substrate. Furthermore, the study revealed that BT-T effectively suppresses the migration of ions under combined light and heat stress. This passivation effect is attributed to the formation of stable hydrogen bonds between BT-T and volatile species, specifically methylammonium (MA⁺) and iodide (I⁻) ions, thereby improving the operational stability of the devices. Consequently, devices incorporating BT-T retained 95.4% of their initial PCE (23.5%) after 1,960 h of continuous MPPT under 1-sun illumination at 65 °C. Tang et al. [90] designed fluorinated SAM molecule 4-(2,7-bis(3-fluoro-4-vinyl phenyl)-9H-carbazol-9-yl)benzoic acid (2,7CZ-FV) with two 4-vinylphenyl terminals to afford thermal cross-linking ability. Cross-linked 2,7CZ-FV formed a uniform and dense interface, which may act as barrier of ion migration. Besides, 2,7CZ-FV can form strong coordination bonds with undercoordinated Pb^2^⁺ in perovskites while simultaneously establishing hydrogen bonds with organic cations. This effectively suppresses non-radiative recombination and ion migration, ultimately leading to significant enhancements in both photovoltaic performance and stability of PSCs. Ultimately, the 2,7CZ-FV-based device achieved a PCE of 24.17%, and retained ∼90% of its initial PCE after 1000 h upon aging at 100 ± 2 °C in a N_2_-filled environment.

## Applications of Self-Assembled Monolayers in Inverted Perovskite Solar Cells

### HSCs

HSCs deposited on substrate are in direct contact with the perovskite layer in IPSCs, determining the efficiency of hole extraction, transfer, and collection as well as the crystallization quality of the subsequent perovskite film. In IPSCs, commonly used hole transport materials such as PEDOT: PSS, PTAA, and NiO_x_ face limitations that restrict further improvements in device performance. For instance, PEDOT: PSS is hygroscopicity, leading to chemical instability upon contact with perovskite precursor solutions, and it exhibits energy-level mismatch compared with other HTLs [122]. Meanwhile, the hydrophobic property of PTAA is unable to provide suitable substrate for growth of perovskite crystal, contributing to poor perovskite morphology [123]. In addition, pristine NiO_x_ requires doping and thermal treatment to improve its hole mobility and film quality. Fortunately, the emergence of self-assembled monolayer-based HSCs demonstrates significant potential to effectively address the aforementioned-challenges.

**Fig. 9:**
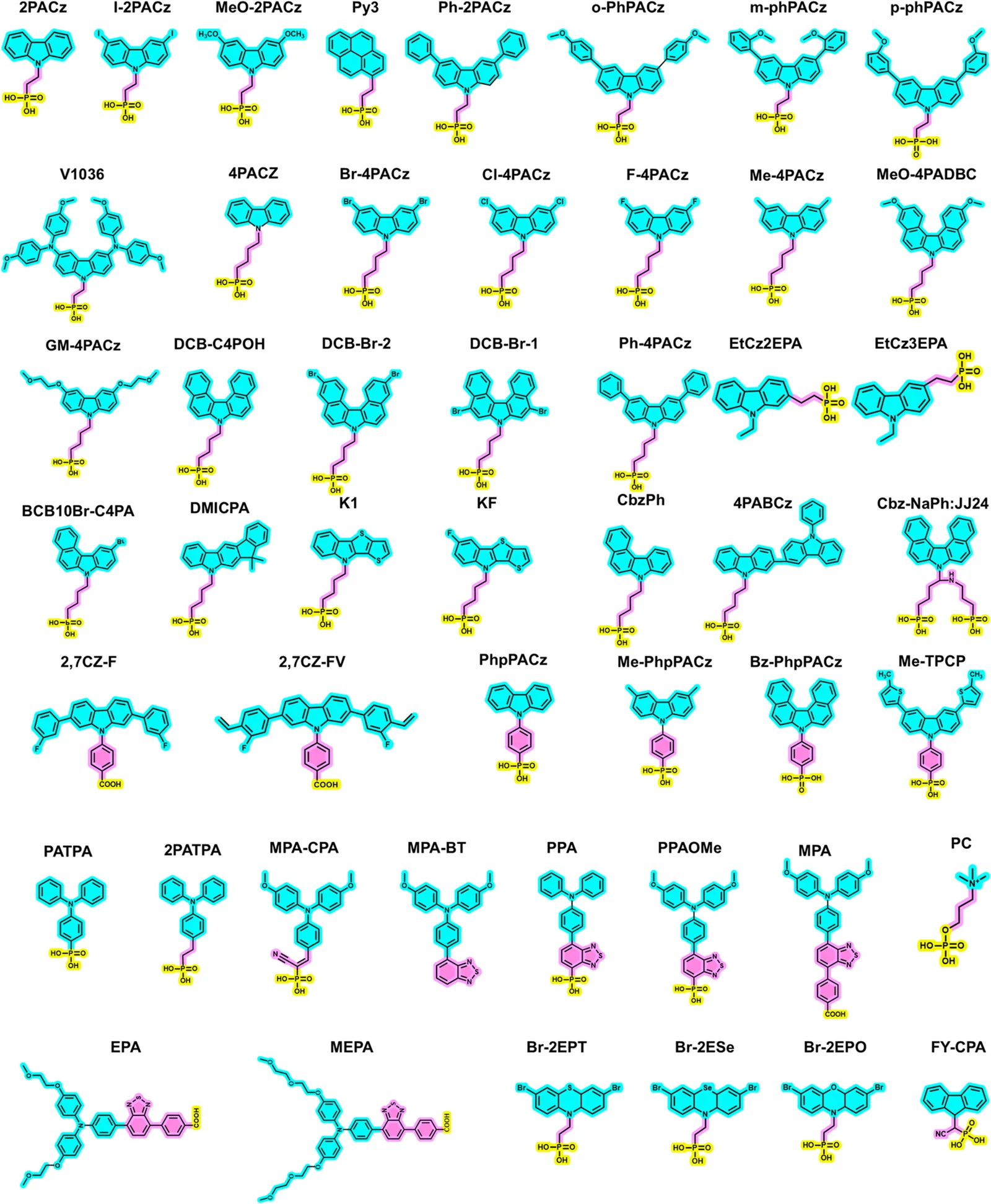
Molecular structure of SAMs with different groups in IPSCs

The widely utilized SAMs as hole-transporting materials in IPSCs can be divided into following species:

(i) Carbazole and its derivatives, equipped with carbazole derivative terminal group, long alkyl chains linker group and phosphonic acid anchoring group. Figure 9 summarizes the chemical structure of typical SAMs in IPSCs utilized as HSCs. Magomedov et al. [2] firstly developed a novel carbazole-derived molecular compound, V1036 ((2-{3,6-bis[bis(4-methoxyphenyl)amino]–9H-carbazol-9-yl}ethyl)phosphonic acid), employed as a HSCs in IPSCs. This material-based device achieved a notable PCE of 17.8%, along with a high average FF of 80%. Afterward, the structure of V1036 was simplified as 2PACz ([2-(9H-carbazol-9-yl)ethyl]phosphonic acid) and MeO-2PACz ([2-(3,6-dimethoxy-9H-carbazol-9-yl)ethyl]phosphonic acid) with reduced absorption in visible light region [8]. Various substituted functional groups (methoxy and methyl) were introduced into carbazole structure to improve hole extraction with perovskite films. Impressively, 2PACz and Me-4PACz ([4-(3,6-dimethyl-9H-carbazol-9-yl)butyl]phosphonic acid)-based HTLs significantly enhanced device performance, particularly in terms of FF. Consequently, the single-junction PSCs achieved an FF of 84% and a certified PCE exceeding 29% for monolithic perovskite/silicon tandem solar cells with assistance of Me-4PACz as the HSC [6].

(ii) Triarylamine (TPA) and its derivatives: A series of heteroatoms such as O, S, or Se have been introduced to precisely regulate molecular energetics and operating characteristics of tricyclic aromatic rings based IPSCs [124]. The rigid carbazole units facilitate a densely packed interfacial configuration, which can generate considerable stress on the perovskite, adversely affecting its structural integrity and threatening interface stability [76]. Conversely, triarylamine structures possess an inherent flexibility that allows for tilting and twisting motions. This mobility provides an effective channel for dissipating stress within the perovskite layer [125, 126]. Furthermore, such flexibility can facilitate the perovskite crystallization process, promoting the formation of high-quality crystals with improved properties [19]. Wang’s group employed a D-A type molecular system (MPA-BT-CA) as an HSC [26]. This novel molecular comprising a CA anchoring group, which not only downshifted the HOMO level but also anchored on ITO substrates through chemical adsorption via the self-assembly method, therefore, passivating surface defects and regulating the ITO work function. Consequently, the fabricated IPSCs achieved a notable PCE of 21.24%, along with enhanced long-term and thermal stability. Xu et al. [9] employed (4-(diphenylamino)phenethyl)phosphonic acid (2PATPA), with flexible triphenylamine head group as HSC. 2PATPA provided planar and compact substrate for perovskite crystals growth, alleviated lattice stress and reduced PbI_2_ defects. 2PATPA-based devices exhibited superior energy-level alignment, enhanced hole extraction, and improved charge transport efficiency, thereby effectively suppressing non-radiative recombination. Consequently, the target devices achieved PCEs of 26.21% and 24.49% for small (0.0715 cm^2^) and large (1 cm^2^) active areas, respectively. Recently, Yip’s group introduced ((2-(4-(diphenylamino)phenyl)-1-phosphonovinyl)phosphonic acid (TPA2P) featuring bisphosphonic acid anchoring group into IPSCs. The bisphosphonic acid anchoring group bonded to ITO through multidentate chelation, significantly enhancing binding affinity, thereby improving SAM layer coverage of substrate. As a result, TPA2P-based devices obtained a champion PCE of over 26%, and maintained 96.7% of their initial value after 1000 h without encapsulation. In contrast, TPAP-based counterparts archived an inferior PCE of 23.88% and retained 85.6% of their initial performance under the same condition [127]. Considering its milder nature, boric acid serves as a complementary anchoring group for SAM-HTMs, providing an alternative to the widely employed carboxylic and phosphonic acid groups [31].

### Interlayers

When employed as the sole HTMs, SAMs may desorb under continuous thermal stress, leading to reduced conductivity and inferior coverage of the hole-selective contact. Construction of NiO_x_/SAMs and SAMs bilayer can address this issue.

#### NiO_x_/SAMs Bilayers

Recently, Wang et al. [87] introduced 2-methoxyethoxy and 2-(2-methoxyethoxy)ethoxy groups into the triphenylamine moiety with polar oligoether chain to produce two novel SAMs, named EPA and MEPA. XPS analysis demonstrated elongation of oligoether chain suppressed Ni^3+^ species, effectively inhibiting Ni vacancies induced degradation of perovskite. While ratio of NiOOH increased on NiO_x_ surface, contributing to enhancement of conductivity of NiO_x_ film. XPS results revealed the phosphate group and Cl^–^ in PC interacted with NiO_x_, as evidenced by a shift of the Ni 2*p* peak toward lower binding energy and the O 1*s* peak toward higher binding energy. XRD patterns revealed the incorporation of larger Cl^–^ ions into the NiO_x_ lattice, as evidenced by the blue shift of the (200) peak, which indicated lattice expansion. Combined with FTIR spectroscopy characterization, they revealed PA groups, quaternary ammonium ions, and Cl⁻ in PC participated in Lewis acid–base interactions with Pb and formed a low-dimensional complex PCPbI_2_. Strong interaction between MEPA and PbI₂ slowed down the crystallization of perovskite, thereby improving its crystallinity and increasing average grain size. Overall, oligoether chain enabled better coverage and strengthened adhesive force between NiO_x_ and perovskite, facilitating bottom-up regulation of perovskite growth and energetics. Consequently, MEPA-based device achieved a champion PCE of 25.50% and a high FF of 85.04%, outperforming EPA (24.92%) and pristine counterparts (24.06%). Simultaneously, MEPA-based device exhibits excellent thermal stability, maintaining 90.3% of its initial PCE after aging at 60 °C for 2500 h without encapsulation. NiO_x_/SAMs bilayer was designed to create strong interface toughening, effectively improving the thermal stability of IPSCs. Carbazole-based SAM (4-(3,11-dimethoxy-7H-dibenzo[c,g]carbazol-7-yl)butyl)phosphonic acid (MeO-4PADBC) was designed to adjust the NiO_x_ HSC/perovskite interface. NiO_x_/SAMs bilayer provided a suitable energy-level alignment with the perovskite layer for hole extraction and constructed strong binding interaction between perovskite and bilayer to resist thermal stress. As a result, the NiO_x_/MeO-4PADBC bilayer-based device obtained a certified PCE of 25.6%, exhibiting dramatically superior operational stability from 25 to 100 °C [23]. Yan et al. [128] adopted phosphorylcholine chloride (PC) together with Me-4PACz to form co-SAM on NiO_x_ surface to passivate NiO_x_ surface defects and fill organic cations and halogen vacancies at the buried interface of perovskite film. XPS results revealed the phosphate group and Cl⁻ in PC interact with NiO_x_, as evidenced by a shift of the Ni 2*p* peak toward lower binding energy and the O 1*s* peak toward higher binding energy. XRD patterns revealed the incorporation of larger Cl⁻ ions into the NiO_x_ lattice, as evidenced by the blue shift of the (200) peak, which indicated lattice expansion. Combined with Fourier transform infrared spectroscopy characterization, they revealed PA groups, quaternary ammonium ions, and Cl⁻ in PC participated in Lewis acid–base interactions with Pb and formed a low-dimensional complex PCPbI_2_. As a result, NiO_x_/co-SAM-based device achieved a champion PCE of 25.09%, and retained 93% of its initial PCE value after continuous illumination for 1000 h.

There are two main challenges associated with NiO, as an HTL are: (1) Ink de-wetting during perovskite deposition causes film shrinkage and pinholes, and (2) the complex redox chemistry of NiO_x_ (Ni^3^⁺/Ni^2^⁺) creates interfacial defects and extraction barriers. These combined effects obstruct the fabrication of continuous, large-area perovskite layers [129–131]. Traditionally, anchoring involves PA groups interacting with the substrate, a configuration known as downward phosphate anchoring (DPA). In this structure, both the PA group and the π-conjugated ring of Me-4PACz exhibited strong adhesion to the NiO_x_ surface, which may induce interfacial disordered arrangement. Liu et al. [132] introduced a Brønsted acid to eliminate hydroxyl groups on NiO_x_, so that the PA group only bonded with the perovskite layer, while the extended π-conjugated ring interacted with the NiO_x_ surface via hydrogen bonds formed between C–H groups in Me-4PACz and NO₃⁻ groups on NiO_x_ surface. This method constructed upward PA anchoring (UPA), which enhanced intermolecular interactions and affinity toward perovskite materials. As a result, UPA-based device achieved a champion PCE of 25.9% with superior illumination, thermal, and humidity stability. The ideal SAMs interlayer should (1) feature strong molecular rigidity and multiple anchoring sites to ensure robust binding to NiO_x_ nanoparticles, (2) adopt a favorable binding geometry and achieve uniform surface coverage to avoid non-ideal contact between NiO_x_ and the perovskite layer, and (3) provide chemical stabilization at the buried interface to minimize defect generation and suppress non-radiative recombination losses. Multidentate anchoring shows effective passivation of Ni species. Wang et al. [133] designed ((9H-fluoren-9-ylidene)methyl) cyanophosphonic acid (FY-CPA) to enhance the interaction with bottom NiO_x_, forming tetradentate binding motifs and constructing a parallel orientation perpendicular to the surface, thereby significantly enhancing charge extraction efficiency. The highest Ni^3+^/Ni^2+^ ratio in FY-CPA-coated NiO_x_, was beneficial for hole transport. Consequently, the target device demonstrated a certified PCE of 25.99%, accompanied by excellent operation and thermal stability. Recently, a novel reductant [9tris(2-carboxyethyl)phosphine hydrochloride (TCEP) was inserted between NiO_x_ and SAMs, in situ forming C–O–Ni coordination bonds and O–H⋯O–Ni hydrogen bonds, along with the connection of –COOH group with the –PO(OH)₂ of SAMs via phosphonate and hydrogen bonding. This intercalated structure strengthens hole extraction and reduces interfacial non-radiative recombination. As a result, the target device achieved an impressive PCE of 26.34%. Moreover, it demonstrates exceptional operational stability, retaining 97.5% of its initial value after 1,000 h under continuous 1-sun illumination, and 90.1% after 1000 h of thermal aging at 85 °C in a nitrogen atmosphere [134].

#### SAMs Bilayers

SAMs molecules with weak binding interaction are prone to elution by polar solvents used in the perovskite deposition process, impairing photovoltaic performance and stability of PSCs. When spin-coating perovskite precursors, DMF solvent in precursor will elute all the upper layer molecules of SAMs, as well as nearly half of the first layer molecules. SAMs suffer from significant structural reconstruction [135].

**Fig. 10:**
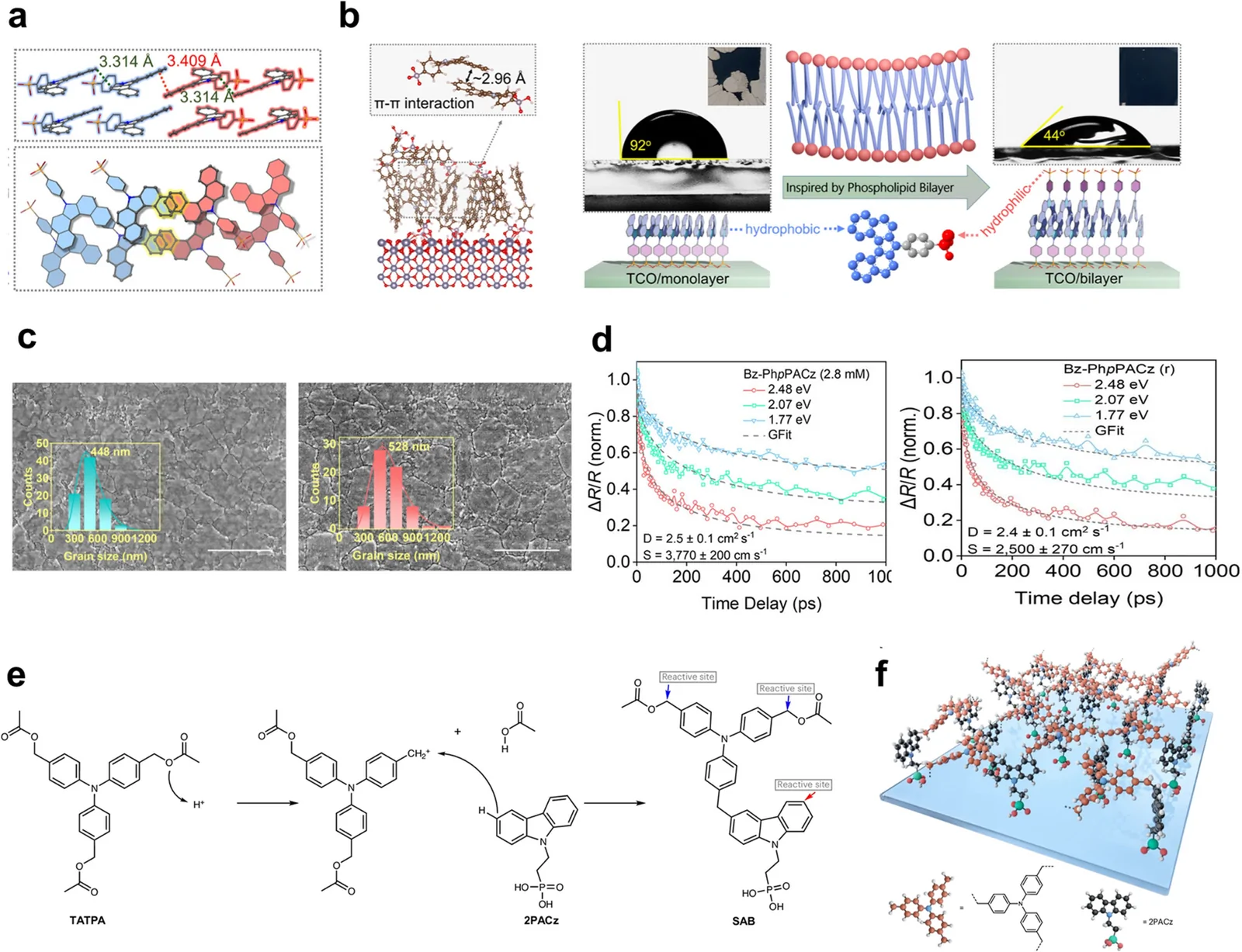
**a** Packing patterns in the single-crystal structure of Bz-PhpPACz. **b** Final configurations of multiple bilayers of Bz-PhpPACz molecules stacking onto the FTO surface after a 10 ps of AIMD simulation. And proposed bilayer structure of Bz-PhpPACz mimicking from phospholipid bilayer and its corresponding contact angles. **c** SEM images and corresponding grain size statistics of the buried surface for the perovskite films deposited on rinsed Bz-PhpPACz (left) and Bz-PhpPACz (right). **d** Surface carrier kinetics probed by TR spectroscopy curves for perovskite deposited on Bz-PhpPACz (left) and rinsed Bz-PhpPACz (right). Reproduced with permission [60]. Copyright 2025, Spring Nature.** e** Proposed mechanism for the coupling reaction between TATPA and 2PACz. **f** Schematic diagram illustrating the structure of SAB. Reproduced with permission [113]. Copyright 2025, Spring Nature

Constructing SAMs bilayer can strength intermolecular interactions and offset the loss of molecules. Gong et al. [135] employed redeposition strategy that new SAM molecules were introduced into the surface of the SAM after being cleaned by DMF to form structural bilayers. Compared with the DMF cleaned samples without redeposition, the molecular retention of the SAMs treated with redeposition increased by 13% to 21% after the second DMF cleaning. The higher coverage and less defects of SAMs after redeposition were beneficial for Cs_0.06_FA_0.94_PbI_3_ perovskite growth and boosted PCE of MeO-2PACz-based device to 22.3% and a fivefold improvement in operational stability compared to unwashed devices. This structural bilayer is observed by Wu et al. [19], they revealed that SAM molecule of MPA-CPA formed a bilayer stack, consisting of a chemically anchored SAM topped by an unadsorbed, disordered overlayer. The unadsorbed amphiphilic MPA-CPA overlayer exhibited superwetting properties toward the perovskite precursor solution, which significantly facilitated perovskite deposition, particularly on larger-area substrates. The bilayer approach can introduce additional molecular wetting layer above the SAM and improve its surface coverage due to its higher surface energy, conformational degrees of freedom and motion capacity. Xu et al. [60] also found 4-(7H-dibenzo[c,g]carbazol-7-yl)phenyl)phosphonic acid (Bz-PhpPACz) formed an ordered hydrophilic bilayer structure—a chemically anchored monolayer plus a non-adsorbed ordered second layer, and PA terminal groups existed at the perovskite burial interface. Single-crystal structure analysis showed that there was a stronger π–π interaction between the Bz-PhpPACz molecules than Bz-4PACz and PhpPACz molecules. The face-to-face distance of Bz-PhpPACz molecules is 3.409 Å (Fig. 10a). Ab initio molecular dynamics (AIMD) simulations further confirmed that multiple Bz-PhpPACz molecules were arranged in parallel on the surface of FTO substrate, with an atomic distance of 2.96 Å, which was a possible driving force for self-assembly (Fig. 10b). The perovskite film deposited on hydrophilic surface of Bz-PhpPACz bilayer structure showed increased grain size, decreased trap density of states and improved crystallinity (Fig. 10c). Transient reflection (TR) spectroscopy analysis of perovskite films deposited on FTO/SAM substrates is shown in Fig. 10d; the Bz-PhpPACz sample exhibited the best carrier extraction ability with a value of 3770 ± 220 cm s^−1^, larger than that of the Bz-PhpPACz sample rinsing by isopropanol thoroughly. This indicated that the bilayer structure enhanced carriers extraction through the Bz-PhpPACz bilayer. As a result, the Bz-PhpPACz bilayer structure enabled the small-area IPSC to achieve a certified PCE of 26.39%, while the larger-area devices (1 cm^2^) attained a PCE of 25.44%.

Desorption and weak interfacial contact of SAMs concern the temperature stability of IPSCs. Liu et al. [113] deposited the alkylating agent 4,4′,4′-tris(acetoxymethylene)triphenylamine (TATPA) onto a 2PACz-modified ITO substrate via solution processing. By utilizing Friedel–Crafts alkylation, they formed a polymer network, thereby achieving the construction of a self-assembled bilayer (SAB) (Fig. 10e, f). The cross-linked polymer network formed by the reaction of TATPA and 2PACz enhanced the overall structural stability of SAB, enabling it to resist thermal degradation at 100 °C for up to 200 h. The planar TPA in SAB presented a surface-oriented adhesive contact with the perovskite surface, significantly enhancing the adhesion energy at the interface. The buried interface of perovskite peeled off from SAB–ITO substrates showed less void formation and fewer impurity phases confined to grain boundaries, suggesting strong interfacial adhesion between the SAB and the perovskite layer. Theoretical calculation revealed that the interaction at the TPA-perovskite interface was stronger, which led to extensive intermolecular charge transfer and enhanced interlayer bonding. As a result, the device PCE based on SAB is as high as 26.3%, which was superior than that of devices based on SAM (24.4%). Furthermore, they exhibited exceptional stability, with efficiency losses below 4% after 2,000 h under 85 °C/85% RH exposure, and below 3% after 1,200 thermal cycles ranging from − 40 to 85 °C [113]. Tang et al. [136] proposed a bilayer SAM (bi-SAM) approach by sequentially assembling Me-4PACz and 3-mercaptopropyltriethoxysilane (MPTS) on NiO_x_. The sequential deposition bi-SAM strategy filled the surface defect of Me-4PACz through the multitooth Si–O–Ni of MPTS, and the -SH in MPTS regulated surface polarity and interacted with uncoordinated Pb^2^⁺, collaboratively optimized the interface energy-level matching and crystallization quality. The optimized IPSC based on bi-SAM achieved a PCE of 25.19%, and maintained 80% of its initial value after stored in air at 60 ± 5% RH for 1400 h without encapsulation, whereas the control device maintained only 35% under the same conditions.

## Large-Area Manufacturing

Despite exciting advancements in the efficiency and stability of SAM-based IPSCs, their large-area scalability remains a major bottleneck, with device performance deteriorating significantly as the active area expands. Dong’s work demonstrated a large decline in PCE occurred when the active area increased from 0.0655 to 749 cm^2^; using the same SAMs, the PCE degraded from 26.2% to 20.21% by using the same SAMs [137]. This decline in PCE is common in solar cell modules [21, 138]. The significant size-dependent PCE loss observed in SAM-based IPSCs may be attributed to scaling issues of the SAM itself and the deposition methods. The film quality of SAMs-coated substrate directly determines the perovskite upper layer and interfacial charge transfer. Deposition methods also significantly impact the distribution of SAMs. Besides, low production cost is a key advantage of PSCs over conventional photovoltaic technologies. To enable large-scale deployment, the material and manufacturing costs of PSCs must be minimized as far as possible. In this part, we aim to expound the challenges of large-scale industrialization of SAMs-based modules and provide insights for its commercialize.

### SAM Aggregation Regulation

Homogeneity and uniformity of the SAMs are crucial for large-scale fabrication. Aggregates may lead to an inhomogeneous substrate surface, thereby adversely affecting the uniformity and crystallinity of the perovskite film growth. According to previous analyses, aggregation behavior can be regulated through molecular design, molecule-solvent and solvent-substrate interaction, and post-processing strategies.

Introducing asymmetric terminal groups can disrupt planarity and reduce aggregation. Tang et al. [139] designed and synthesized 4-(10-bromo-7H-benzo[c]carbazol-7-yl)butylphosphonic acid (BCB10Br-C4PA), an asymmetric conjugated SAM functionalized with a terminal bromine substituent. BCB10Br-C4PA yielded a large dipole moment and high solubility, which could decrease micelle formation and increase coverage and continuity of the SAMs. Polar terminal groups or asymmetric charge distributions strengthen dipole–dipole interactions and promote the formation of interfacial dipoles. This not only enables more uniform molecular packing but also optimizes energy-level alignment at the interface. He et al. [79] developed a π-conjugation extended carbazole derivatives, 7,7-dimethyl-5,7-dihydroindeno[2,1-b]carbazole (DMICPA), in which two methyl groups attached to the asymmetric indene structure effectively suppressed molecular aggregation. Compared to the symmetric counterpart 4PACz, this asymmetric π-conjugation extension provided improved energy-level alignment, enhanced dipole moments, and stronger interactions with the perovskite layer. Consequently, the DMICPA-based device delivered a champion PCE of 26.27% and exhibited excellent operational stability, retaining over 90% of its initial PCE after 1,000 h under MPPT condition. Introducing hydrophilic or steric hindrance side chains can regulate the intermolecular interactions to inhibit undesired self-aggregation in solution or during film formation. A glycol monomethyl ether was introduced into 4PACz, yielding a new SAM abbreviated as GM-4PACz. GM-4PACz had a larger polarity and better solubility in ethyl alcohol, reducing aggregates in solution. The inclusion of GM groups elevated the surface energy of the ITO/SAM substrate, thereby promoting the nucleation and growth of the overlying perovskite film. This modification also suppressed cation defects, alleviated residual stress at the SAM/perovskite interface, and optimizes the energy-level alignment for improved compatibility with the perovskite layer. Consequently, the device based on GM-4PACz achieved a champion PCE of 25.52% with a *V*_OC_ of 1.21 V and high stability, retaining 93.29% and 91.75% of its initial value after aging in air for 2,000 h or under MPPT for 1,000 h, respectively [140].

The polarity of the solvent governs the aggregation configuration of SAM molecules, while the dielectric constant of the solvent, which reflects its coordination strength with the substrate, determines whether SAM molecules can effectively access the substrate [141, 142]. Gooding et al. [141] studied packing behavior of phosphonic acids on indium tin oxide (ITO) surfaces from different solvents (triethylamine, ethyl ether, tetrahydrofuran (THF), pyridine, acetone, methanol, acetonitrile, dimethyl sulfoxide (DMSO), or water). The dielectric constants of solvents are shown in Table 1 from Gooding’s research. They demonstrated that higher dielectric constants (DCs) of the assembly solvents led to lower molecular surface density, increased defect formation, and reduced robustness of the resulting SAMs. These adverse effects were more pronounced in aromatic systems than in long-chain aliphatic SAMs. Notably, pyridine served as a striking exception, regardless of the molecular system, SAMs assembled from pyridine exhibited the poorest quality by a substantial margin. They maintained the belief that the different solvents influenced the bond formation instead of the packing of molecules on the surface. The stronger the solvent polarity, the greater its adsorption heat on the ITO surface, making it more difficult for phosphonic acid molecules to displace the already adsorbed solvent molecules [141]. The solvent-SAM interaction also plays a pivotal role. Kirchartz et al. [75] demonstrated through computational analysis that NMP exhibited strong binding affinity to both Me-4PACz and perovskite precursors. By employing a ternary solvent system composed of *N*,*N-*dimethylformamide (DMF), DMSO, and NMP, enhanced film uniformity and coverage were achieved. This approach delivered PCEs, exceeding 20% for small-area devices and maintained over 18% for large-area modules. Considering large-scale production and environment factors, green solvents such as isopropyl alcohol (IPA), ethanol, with DCs of approximately 18 and 28, respectively, are promising candidates [143–145].

**Table 1:** DCs of common solvents in SAMs [141]

Solvents	Ethyl Ether	THF	Pyridine	Acetone	Methanol	Acetonitrile	DMSO	Water
DCs	4.22	7.39	12.3	20.5	32.6	37.5	48.9	78.5

Wang et al. [146] demonstrated that spin coating, a SAM solution onto ITO, without any post-treatment (such as solvent rinsing) resulted in a multilayer structure. The bottom layer tended to be orderly arranged due to covalent interactions between the phosphate groups and ITO, while the upper layers exhibited a disordered arrangement. In contrast, the ITO/SAM layer treated with methanol rinsing formed a true monolayer. To ensure the formation of a uniform monolayer and prevent aggregation, an additional post-treatment is needed. A solvent rinsing step is necessary after the initial coating or solution incubation to remove excess molecules. It is essential to thoroughly eliminate physically adsorbed or incompletely reacted SAM molecules to avoid multilayer adsorption or interfacial contamination. IPA effectively dissolves residual unassembled molecules (such as thiol or silane monomers), environmental organic contaminants (e.g., oils and dust), and solvent residues left from SAM deposition. Its low surface tension allows IPA to penetrate the intermolecular gaps within the SAMs, where it loosens and removes impurities through intermolecular forces, such as van der Waals interactions and hydrogen bonding, without disrupting the covalently bonded primary structure of the SAMs. Other commonly used solvents include ethanol, toluene, and hexane. Typical cleaning methods involve soaking, spraying, and ultrasonic-assisted cleaning.

### From Small Molecule SAMs to Poly-SAMs

Recently, poly-SAMs have attracted significant interest because their strong covalent bonds or intermolecular interactions form a cross-linked network, resulting in a more robust structure that is less prone to desorption or rearrangement due to thermal fluctuations, solvent erosion, or external forces [36, 91]. The multipoint anchoring and network structure of polymeric SAMs enable more uniform dispersion of interfacial stress, enhance mechanical coupling with adjacent material layers, and are suitable for large area and flexible devices or brittle interfaces [38].

Chen et al. [38] developed poly-DBPP, which featured an indoloindole core and novel bisphosphonic acid groups. This design enabled simultaneous anchoring to the underlying conductive substrate and effective interaction with the perovskite layer. In situ absorption measurements during ink drying and film crystallization confirmed the slower crystallization on DBPP and poly-DBPP relative to 4PACz, which was attributed to the interaction between their phosphonic acid groups and Pb^2^⁺ cations. This retardation of crystallization ultimately enhanced the film crystallinity. Ultimately, strong interfacial interactions of SAMs ultimately improved photovoltaic performance, yielding a champion PCE of 25.1%, along with good thermal and light stability. As mentioned above, the uniformity and high coverage of the SAMs are related to their thickness. Increasing the thickness of HSCs can partially alleviate this issue, but at the expense of device efficiency and scalability in manufacturing. This demonstrates a fundamental trade-off between stability and efficiency in devices using conventional small-molecule SAMs: Their insulating nature causes PCE to degrade rapidly as SAM thickness increases. Therefore, the same group subsequently developed poly-DCPA and poly-PhPACz to maintain good adhesion on substrate, wettability suitable for perovskite growth in ultrathin thickness [35, 36]. Poly-DCPA, with its better wettability on ITO and higher conductance, promoted ink spreading and interfacial charge transfer. After optimization, ambient blade-coated IPSCs utilizing poly-DCPA (thickness ~ 7 nm) achieved a remarkable PCE of 24.9%, remaining 94% of the initial PCE after over 900 h of light soaking at 85 °C. The DCPA with optimal thickness of 1–2 nm based counterparts exhibited a rapid decline, with over 30% degradation in performance within 400 h [35]. The high sensitivity of these materials to layer thickness and surface coverage results in a narrow processing window and poses significant challenges for achieving uniformity across large-area substrates. Poly-SAMs are suitable for large-scale deposition. On this basis, Chen et al. [36] fabricated ambient blade-coated poly-PhPACz-based PSCs, which achieved an efficiency of 26.1% while maintaining high performance across varying thicknesses and retain 92% of their initial performance after 1800 h of light soaking.

### Deposition Techniques Optimization

**Fig. 11:**
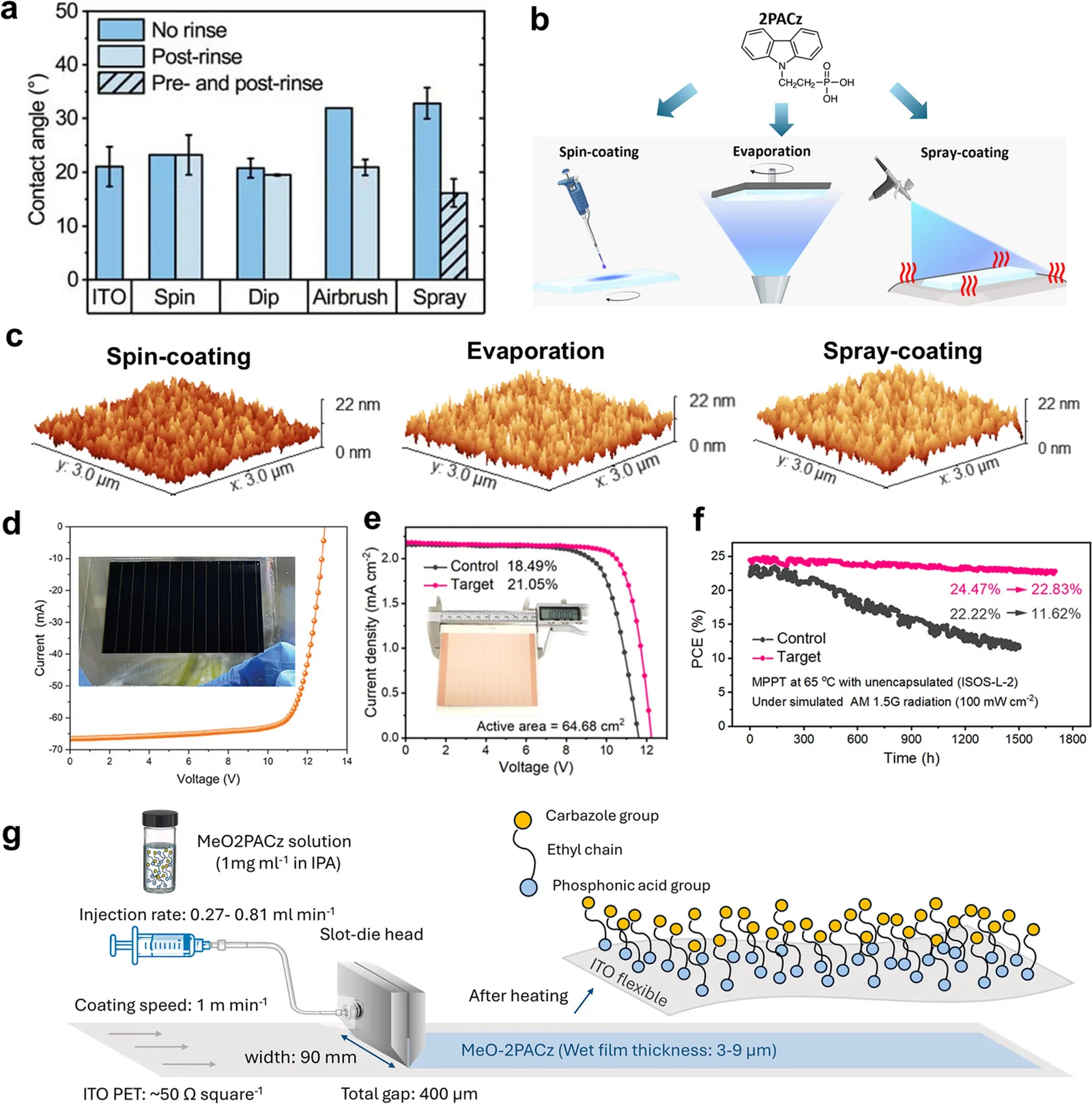
**a** Contact angle for 2-methoxy ethanol (2-ME) on clean ITO and MeO-2PACz films deposited on ITO via each technique without rinsing and for post-rinsed films. Reproduced with permission [147]. Copyright 2022, Wiley–VCH. **b** Schematics of the deposition and optimization methods for 2PACz using spin coating, evaporation, and spray coating. Reproduced with permission [85]. Copyright 2024, Wiley–VCH. **c** Surface roughness of optimized 2PACz layers deposited by spin coating, evaporation, and spray coating. Reproduced with permission [85]. Copyright 2024, Wiley–VCH. **d** I-V curve of champion PSCs (30.03 cm^2^) based on Poly-DBPP. Reproduced with permission [38]. Copyright 2024, Wiley–VCH.** e** J-V curves of the champion devices with NiO_x_/Me-4PACz and NiO_x_/Arg-Me as HTLs. **f** MPP tracking curves of the unencapsulated PSCs with different HTLs measured at 65 °C. Reproduced with permission [21]. Copyright 2025, Wiley–VCH. **g** Schematic of the slot-die coating process for MeO-2PACz solution, incorporating optimized parameters. Reproduced with permission [150]. Copyright 2025, Elsevier Ltd

Solutions-based spray coating and airbrush coating were initially introduced as scalable methods for SAM deposition. Building on this, Lidzey and colleagues advanced ultrasonic spray-coating and airbrush-coating techniques, incorporating optimized solvent rinsing protocols to deposit uniform MeO-2PACz films. After combining the prerinsing and post-rinsing processes, among the MeO-2PACz films prepared by various methods, the spray-coated version exhibited the smallest contact angle with perovskite precursor ink, outperforming those fabricated via spin coating, dip coating, and airbrush coating (Fig. 11a). This indicated superior wettability of the perovskite ink on the spray-coated SAM. The enhanced wettability enabled the deposition of high-quality spray-coated perovskite films, leading to the fabrication of IPSCs with PCEs exceeding 20% [147]. A solvent-free evaporation method was also developed to fabricate 2PACz HSCs in IPSCs, Qi et al. [85] compared the photovoltaic properties of SAMs processed by spin-coating, evaporation, and spray-coating methods (Fig. 11b). In thermal evaporation process, they found incorporating thicker 2PACz layers followed by post-deposition treatment led to enhanced device performance. The average PCEs measured 7.9%, 13.3%, 19.3%, and 17.0% for thicknesses of 1, 10, 50, and 100 nm, respectively, demonstrating the benefit of optimized layer thickness in conjunction with post-processing. During spray coating, the airbrush was held approximately 15 cm away from the substrate while dispensing the ink. The thickness of SAMs was controlled by spraying cycles. The morphology of the 2PACz layers deposited via spin-coating and optimized evaporation and spray coating showed negligible differences, with root mean square (RMS) roughness of 2.48, 2.93, and 2.95 nm, respectively (Fig. 11c). Similarly to thermal evaporation, *V*_OC_ and *J*_SC_ of devices improved with increased thickness of 2PACz, owing to enhanced coverage on ITO. The spray-coated SAMs-based devices achieved an average PCE of 20%. By employing spray coating, they further fabricated devices with an active area of 22.4 cm^2^, achieving a PCE of 13.34%. However, conventional spin-coating method processed devices yielded PCE of 19.7%. It is noted that the optimized morphology of SAMs processed by the three methods showed negligible differences, indicating good coverage of SAMs on ITO.

Recently, blade-coating method has been adopted to fabricate large-scale SAMs. Chen’s group fabricated a poly-DBPP-base perovskite module via blade coating to deposit both SAMs and perovskite layer. Blade coating operates without vacuum or intricate spray systems—only a blade and a flat coating platform are needed. As a result, the perovskite module fabricated via blade coating achieved an impressive PCE of 22.0% at an aperture area of 30.03 cm^2^ (Fig. 11d) [38]. Subsequently, the same group fabricated poly-PhPACz-based devices via blade coating, achieving a PCE of 24.4% on devices with an active area of 1 cm^2^ [36]. Shi et al. [21] compared spin-coating and blade-coating processed SAMs (Me-4PACz), arginine (Arg) molecules were preanchored on NiO_x_ acting as steric barriers to suppress the self-aggregation of SAM. SAMs assisted by Arg preanchored films showed good uniformity on both small-area substrates processed by spin-coating and large-area (10 × 10 cm^2^) substrates fabricated by blade coating. As shown in Fig. 11e, the resulting PSC achieved impressive PCEs of up to 26.67% for small-area devices (active area: 0.049 cm^2^) and 21.05% for large-area modules (active area: 64.68 cm^2^). Remarkably, the devices also demonstrated outstanding operational stability, maintaining 93% of their initial PCE after 1,700 h of MPPT under the ISOS-L-2 testing protocol (Fig. 11f).

Slot-die coating has gained recognition as a highly promising deposition technique, owing to its seamless compatibility with roll-to-roll production, minimal material waste, and excellent controllability over film thickness and crystallization kinetics [148, 149]. Prior research demonstrated that slot-die coating could produce uniform and high-quality perovskite layers on par with spin-coated films, positioning it as a promising approach for scalable module manufacturing. Recently, Waston et al. [150] demonstrated that slot-die coating is capable of fabricate uniform SAMs. The schematic of the slot-die coating parameters is shown in Fig. 11g. The SAM solution is subjected to ultrasonic treatment and filtration to promote the dispersion and alignment of SAM molecules. Slot-die coating produced a uniform SAM layer with a thickness ranging from ∼4.67–7.20 nm over an 18 cm substrate. As a result, the slot-die coated device achieved a PCE of 15.04%.

While numerous scalable deposition techniques have been developed for producing large-area SAM films, several challenges persist in scaling up solar modules that employ SAM-based HSCs. For instance, thermally evaporated SAMs exhibit a significant shift in Fermi level compared to their spin-coated counterparts [85]. This discrepancy was attributed to potential alterations in the chemical properties or thermal degradation of the SAMs during the evaporation process. In addition, ambient exposure and substrate characteristics have a significant impact on the adsorption orientation, and the work function of evaporated SAMs. Consequently, a deeper understanding of the surface properties of evaporated SAMs is critically needed. Furthermore, compared to spin coating, SAMs processed by spray coating and airbrush coating which lack centrifugal force tend to agglomerate and form inhomogeneous film. To mitigate these issues, we suggest that incorporating co-assembling additives or fine-tuning the solvent evaporation rate of precursor solutions could serve as viable strategies. For blade coating, significant challenges remain in achieving uniform film thickness, controlling solvent evaporation, and ensuring process reproducibility. Further optimization of both processing techniques and material systems is still needed for their application. In the future, limitations could be mitigated by improving blade design (e.g., using flexible blades), introducing atmosphere-controlled drying systems, and developing in situ monitoring and feedback technologies. For the slot-die coating method, enhancing its compatibility with low-viscosity and low-concentration SAM solutions is critically needed. This requires the development of more precise pumping and flow-control systems to prevent defects such as “leakage” or “line-break” during deposition.

### Co-SAM Strategy

**Fig. 12:**
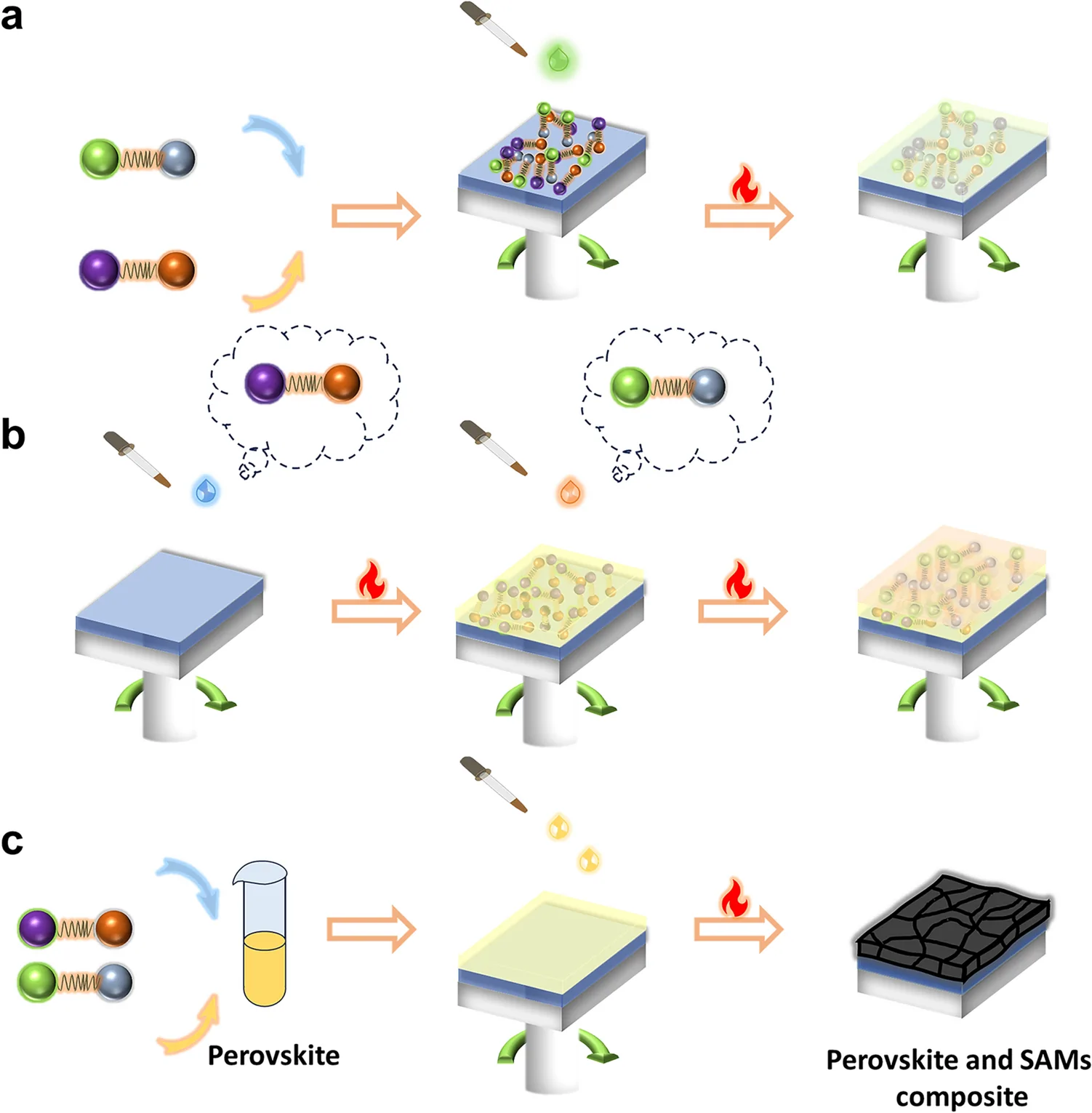
Schematic diagram of co-SAMs: **a** co-adsorption, **b** sequential adsorption, and **c** co-deposition with perovskite

Co-SAMs combine the advantages of two or more kinds of SAMs, aiming to address the present challenges of single SAM such as incomplete coverage, weak bonding with substrate or perovskite, instability, etc. co-SAMs strategy can be realized via three methods simply called co-adsorption, sequential adsorption, and co-deposition with perovskite. Figure 12 schematically illustrates these methods.

#### Co-Adsorption

Co-adsorption is a straightforward process in which two SAMs are dissolved in a solution and simultaneously deposited onto a substrate, forming a mixed SAM through a competitive adsorption process. The final properties of a co-SAM depend on the intrinsic properties of their molecular constituents, their interactions, and their mixing ratio on the surface (Fig. 12a) [151]. Recently, Jen et al. [1] developed an azide-containing cross-linkable guest SAM (JJ24) capable of forming strong covalent bonds with the nearby alkyl linkers from the host SAM (CbzNaph) to form cross-linked co-SAM (CbzNaph: JJ24), as shown in Fig. 12. Co-SAMs perfectly covered the substrate and retained an orientation perpendicular to the substrate. Moreover, the molecular dipole moment of the co-SAM is enhanced, resulting in a down-shift of the work function of the transparent conducting oxide substrate. This modification significantly improves interfacial hole extraction. As a result, the champion IPSCs incorporating this cross-linked co-SAM attained a certified PCE of 26.92%. In the sequential adsorption method, the primary SAM is deposited onto a metal oxide substrate, forming a loosely packed layer, followed by the secondary SAM (Fig. 12b). Compared to the co-adsorption approach, sequential adsorption effectively circumvents unintended segregation or premature interactions between dissimilar SAMs in solution. Furthermore, the use of lower precursor concentration in sequential adsorption mitigates molecular aggregation and promotes the formation of a more uniform co-SAM [113, 152]. SAMs could be directly dissolved in the perovskite precursor solution and subsequently deposited on substrate, called co-deposition. Owing to their relatively low solubility, the SAM molecules precipitate prior to the perovskite components, and preferentially anchor onto the ITO substrate [153]. This approach effectively avoids the hydrophobic nature of preformed SAMs, thereby significantly enriching the varieties of SAMs applicable to PSCs.

#### Sequential Adsorption

The sequential adsorption method has unique characteristics. Conventional amphiphilic SAM molecules (e.g., 2PACz, MeO-4PACz) are inclined to aggregate into clusters, which would be washed away in the following step instead of anchoring on ITO, thus leading to a patchy SAM with bare ITO regions underneath [18]. The secondary SAM could interact with the –PO(OH)_2_ group present in conventional SAMs to competitively inhibit their aggregation and promote uniform dispersion of the primary SAM. Thus, sequential adsorption aims to address the aggregation issue of single SAM. In a pioneering effort to mitigate SAM aggregation via a co-adsorbed molecular strategy, Park and colleagues incorporated 3-mercaptopropionic acid (3-MPA), featuring a thiol (–SH) and –COOH functional group to co-assemble with the conventional 2PACz. –SH is capable of interacting with the –PO(OH)₂ in 2PACz, thereby disrupting its inherent clustering behavior. Simultaneously, –COOH group provides additional anchoring sites to the substrate, enhancing the stability of the molecular assembly [154]. As reported before, trifluoromethyl groups (–CF_3_) could interact with –PO(OH)_2_ groups in 2PACz via hydrogen bonds, which confined the mobility of 2PACz and alleviated the cluster aggregation [76]. Similarly, 4,4′,4″-nitrilotribenzoic acid was introduced to Me-4PACz SAM by competing with Me-4PACz in tetramer formation, inhibiting cluster development and preventing agglomeration. Consequently, the NA/Me-4PACz co-SAM enabled an inverted PSC to achieve a certified steady-state PCE of 26.54% [77]. An ideal underlying surface for perovskite growth should possess good wettability, appropriate surface energy, and a smooth, defect-free morphology. The commonly used SAMs such as 2PACz and Me-4PACz generally show poor wettability, which seriously inhibits the adhesion of perovskite precursor and adversely affects the quality of subsequent perovskite film. Thus, the secondary SAM could also introduce functional groups to bond with perovskite and enhance the interfacial contact. Ashouri et al. [155] added 1,6-hexylenediphosphonic acid (6dPA) into Me-4PACz for improved wettability. Li et al. [76] employed a co-adsorption strategy by introducing 2-chloro-5-(trifluoromethyl)isonicotinic acid (PyCA-3F) in conjunction with the conventional anchor 2PACz. Employing MeO-2PACz, which features a hydrophilic methoxy group, as the secondary SAM yielded consistent outcomes, corroborating the reliability and broad applicability of this molecular design strategy [156–159].

#### Co-Deposition

The co-deposition of SAMs with perovskite precursor is compatible with different molecules, perovskite compositions, solvent systems, and coating methods (Fig. 12c). Li’s group designed a novel p-type molecule (7H,7′H-[5,5′-bidibenzo[c,g]carbazole]-7,7′-diylbis(butane-4,1-diyl)) bis(phosphonic acid) (D4PA), which was mixed with perovskite precursor solution. The molecular structure of D4PA enables homogeneous and strong adhesion to the perovskite and substrate via its intramolecular C–C bonds. Importantly, its inherent steric hindrance prevents molecular aggregation, thereby prolonging the lifespan of precursor solution and ultimately improving device reproducibility. As a result, the target device achieved a certified PCE of 26.72% with outstanding stability by maintaining over 97.2% of its initial performance after 2500 h of continuous operation at MPPT [34]. He et al. [160] developed a molecular complementary passivation (MCP) strategy by introducing propylphosphonic acid 3-ammonium bromide (PPAABr) alongside phenethylammonium bromide (PEABr) into the perovskite precursor solution. This approach effectively passivated bulk defects within FAPbI₃ perovskite crystals. Their results demonstrated PPAA^+^ tended to occupy FA^+^ vacancies in a manner similar to PEA^+^, passivating V_I_ defect states, decreasing interfacial defect density, and improving exciton and carrier lifetime of the perovskite film. Moreover, MCP strategy upshifted the E_F_ of the perovskite layer, matching well with PC_61_BM. Consequently, the target IPSC achieved a champion PCE of 26.40%. However, Chen et al. [36] found that while co-deposition could simplify the fabrication process, the resulting devices exhibited a markedly lower reverse breakdown voltage (V_rb_) compared to those processed by sequential adsorption, a drawback that may limit their industrial applicability. This anomaly may be attributed to the high dipole moment of poly-SAMs, which strengthens their interaction with polar solvents and thereby suppresses the separation and predeposition of SAMs. Mazzarella et al. [161] found MeO-4PACz had a lower dipole moment than the other SAMs molecules, potentially weakening its interaction with the DMF:DMSO solvent. This enables it to separate more rapidly from the solvent during annealing, thereby promoting the formation of a distinct layer adjacent to the perovskite rather than remaining dispersed within the perovskite bulk layer.

### Cost Analysis of SAMs

Inherently low production cost of PSCs distinguishes it from traditional photovoltaic technologies. Achieving widespread commercial adoption, however, necessitates further reduction in both material and manufacturing expenses, while the perovskite active layer itself can be fabricated at minimal cost. Due to complex synthesis and purification procedures, the price of hole-selective materials, particularly SAMs, remains expensive [162]. For example, the synthesis of commonly used p‑type polymer materials, including PTAA, PEDOT:PSS, and poly‑TPD, requires precise control of molecular weight and often involves expensive catalysts [163]. Although the synthesis of widely employed SAMs, such as 2PACz and MeO-2PACz, demands the same complex reaction conditions, their raw materials or intermediates have already been established through prior research in organic light-emitting diodes and organic photovoltaics [164, 165]. Moreover, the material consumption of SAMs in fabricating IPSCs is significantly lower than that of PTAA and NiO_x_ counterparts, owing to their monolayer-level thickness. As shown in Table 2, previous studies indicate that the unit material cost of 2PACz SAM is markedly lower (0.041 USD) than PTAA (2.424 USD), poly‑TPD (0.529 USD), PEDOT:PSS (0.224 USD), and NiO_x_ (0.068 USD) [165]. The unit material cost is estimated based on the material expenditure required to fabricate a single device on a 2 × 2 cm^2^ substrate, assuming solution‑based spin‑coating as the deposition method for all HSCs. Facility‑related expenses associated with the deposition process such as electricity consumption and labor costs are excluded from this analysis. All material prices (in US dollars) were sourced from websites of chemical manufacturers, and the compiled data are summarized in Table 2.

**Table 2:** Cost analysis of the HSCs for fabricating one piece of device [165]

HSC		Using amount	Price (USD)	Cost	Concentration
PTAA		0.5 g	474.6 (0.1 g)	2.424	5 mg mL^−1^ (toluene)
NiO_x_	NiO_x_(Ni(HCOO)_2_ ·2H_2_O)	9.2 g	37.8 (100 g)	0.068	92 mg mL^−1^ (ethylene glycol)
	Ethylenediamine	0.0033 mL	31.0 (5 mL)		
PEDOT:PSS AI4083		0.1 mL	224.2 (100 mL)	1.5 wt% (water)	1.5 wt% (water)
Poly-TPD		1.0 mg	475.4 (1.0 g)	0.529	10 mg mL^−1^ (chlorobenzene)
2PACZ		0.1 mg	192.6 (0.5 g)	0.041	1 mg mL^−1^ (ethanol)

With the continuous advancement of the photovoltaic industry, the pursuit of higher efficiency and stability advances the development of highly complex molecular structures. Those structures—often featuring polycyclic frameworks, asymmetric designs, or multiple functional groups—enable more precise control over energy levels, molecular orientation, and interfacial interactions, thereby enhancing device efficiency and stability. However, such structural complexity often entails multistep synthesis, low yields, difficult purification processes, and expensive raw materials or catalysts, which significantly increase production costs and hinder scalability. In the early research and development stage, a certain degree of molecular complexity can be tolerated to explore performance limits, but synthesis cost data (e.g., $ g^−1^, number of steps, yield) should be documented concurrently. For industrial-scale implementation, the cost-performance ratio must be a central criterion in molecular design. This calls for technological innovations that achieve high performance with simpler structures. For instance, compensating for molecular-level limitations through advanced interface engineering. In total, the authors maintain that an optimal molecular design must balance efficiency and cost, either achieving a significant performance leap without a substantial rise in expense, or preserving the majority of performance benefits through intelligently simplified molecular architecture.

## Summary and Outlook

In conclusion, SAMs based on electron-rich conjugated backbones and phosphonic acid anchoring group functionalities have been successfully applied in high-performance IPSCs. The PCE has boosted from 17.8% in 2018 to the current 26.9%, through improving the energy-level match, reducing defect densities, enhancing the interfacial charge transfer efficiency, and inhibiting ion migration. The photovoltaic performance parameters and stability testing results of typical SAMs-based IPSCs are summarized in Table 3. We discussed their components and analyzed the relationship between structure and properties (solubility, conductivity, surface state, and packing behavior). SAMs can serve not only as hole-selective contacts but also interface modifiers, which provide new opportunities for economical, scalable, and stable IPSCs.

**Table 3:** Photovoltaic parameters and stability of SAMs-based IPSCs recently reported

SAMs	Active layer	Performance				Stability		References
		*J*_SC_ (mA cm^−2^)	*V*_OC_ (V)	FF (%)	PCE (%)	Retention rate (%)	Testing condition	
V1036	Cs_0.15_FA_0.65_MA_0.2_Pb(I_0.8_Br_0.2_)_3_	21.20	1.04	79.3	16.9	> 88	40 °C, N_2_, 11 h (MPPT)	[8]
MeO-2PACz		22.20	1.14	80.5	20.2	> 97		
2PACz		21.90	1.19	80.2	20.8	> 97		
Ph-2PACz		20.50	1.26	82.6	21.3	> 90	25 °C, encapsulated, 680 h (MPPT)^*a*^	[7]
PPAOMe	Cs_0.05_FA_0.85_MA_0.1_PbI_3_	24.7	1.10	79.2	21.52	< 70	55 ± 5 °C, N_2_, 1000 h (MPPT)	[114]
Me-4PACz		25.23	1.188	81.3	24.14	> 74	50 ± 10 °C, N_2_, 3000 h (MPPT)	[56]
Me-Ph*p*PACz		25.37	1.19	86.8	26.17	> 98		
PATPA		25.85	1.19	85.5	26.21	~ 91	40 ± 10 °C, N_2_, 1000 h (MPPT)	[9]
Ph*p*PACz		25.27	1.16	80.8	23.67	~ 65		
2PATPA		25.70	1.17	83.8	25.29	~ 71		
CBzNaPh		25.91	1.17	85.2	25.86	~ 80	85 °C, RH^*b*^ 70–80%, encapsulated, 450 h (MPPT)	[1]
CBzNaPh:JJ24		26.18	1.18	86.7	26.92	~ 100	85 °C, RH 70–80%, encapsulated, 1000 h (MPPT)	
4PADCB		25.26	1.186	84.27	25.25	< 30	50 C ± 5 °C, N_2_, unencapsulated, 1000 h (MPPT)	[91]
4PADCB-V		25.41	1.184	84.57	25.45	< 50		
crs-4PADCB-V		25.68	1.194	86.29	26.46	91		
2PACz	(FA_0.98_MA_0.02_)_0.95_Pb(I_0.98_Br_0.02_)_3_	25.70	1.140	80	23.1	~ 45	55 °C, N_2_, 600 h (MPPT)	[17]
Py3		26.00	1.180	85.1	25.9	> 99		
CbzNaph	Cs_0.05_MA_0.15_FA_0.80_PbI_3_	24.69	1.170	83.4	24.1	> 97	25 °C, N_2_, continuous illumination for 120 h	[24]
MPA-BT	(FA_0.17_MA_0.94_PbI_3.11_)_0.95_(PbCl_2_)_0.05_	21.96	1.090	76.6	18.34	~ 70	25 °C, H 72%, encapsulated, stored for 14 d	[26]
MPA-BT-CA		22.25	1.130	84.8	21.24	> 98		
4PACz	Rb_0.05_Cs_0.05_MA_0.05_FA_0.85_Pb(I_0.95_Br_0.05_)_3_	24.91	1.15	81.3	23.31	~ 68	60 ± 5 °C, RH 50 ± 5% encapsulated, dark condition after 645 h of storage	[103]
K1		25.17	1.17	83.6	24.66	~ 76		
KF		25.33	1.19	84.3	25.35	~ 80		
Br-2EPO	FA_0.92_MA_0.08_Pb(I_0.92_Br_0.08_)_3_	24.25	1.07	80.7	21.02	~ 33	30 °C, RH 15%-25%, encapsulated, 500 h (MPPT)	[124]
Br-2EPT		24.41	1.09	81.3	21.63	> 80		
Br-2EPSe		24.49	1.12	82.9	22.73	> 96		
TPAP	Cs_0.05_FA_0.95_PbI_3_	25.09	1.17	81.6	23.88	> 96.7	40 °C ± 5 °C, N_2_, 1000 h (MPPT)	[127]
TPA2P		25.99	1.18	85.0	26.11	> 96.7		
NiO_x_/MeO-4PADBC	Cs_0.15_FA_0.85_Pb(I_0.6_Br_0.4_)_3_	25.40	1.16	82.1	21.6	> 96	25 °C, encapsulated, under constant 1-sun illumination of 1200 h	[23]
NiO_x_/MPA	Cs_x_FA_1-x_PbI_3_	25.53	1.144	82.9	24.06	> 84.1	60 °C, N_2_, stored for 2500 h	[87]
NiO_x_/EPA		25.39	1.17	84	24.92	> 89.5		
NiO_x_/MEPA		25.63	1.17	85	25.50	> 90.3		
Me-4PACz /MPTS	Cs_0.05_FA_0.85_MA_0.1_PbI_3_	25.34	1.159	85.75	25.19	80	RT, RH 60 ± 5%, unencapsulated, stored for 1400 h	[136]
MPA-CPA bilayer	Cs_0.05_(FA_0.95_MA_0.05_)_0.95_Pb(I_0.95_Br_0.05_)_3_	24.80	1.21	84.7	25.4	~ 100	45 °C, RH 30–40%, encapsulated, 500 h (MPPT)	[19]
Bz-PhpPACz bilayer	Cs_0.03_FA_0.97_PbI_3_	26.17	1.185	85.11	26.39	~ 100	65 ± 10 °C, N_2_, unencapsulated, 3000 h (MPPT)	[60]
SAB	Cs_0.05_(FA_0.95_MA_0.05_)_0.95_Pb(I_0.95_Br_0.05_)_3_	26.2	1.17	85.5	26.3	> 96	85 °C, RH 85%, encapsulated, stored for 2000 h	[113]
GM-4PACz	Cs_0.05_(FA_0.95_MA_0.05_)_0.95_Pb(I_0.95_Br_0.05_)_3_	25.43	1.21	82.75	25.52	91.75	The same condition, 1000 h (MPPT)	[140]
Me-4PACz		25.10	1.16	80.17	23.28	91.75	RH 30 ± 5%, encapsulated, 586 h (MPPT)	
MeO-4PACz		25.00	1.13	80.12	22.54	52.9	The same condition, 878 h (MPPT)	
Me-4PACz		25.24	1.175	81.39	24.14	80	40 °C, N_2_, under constant 1-sun illumination for 500 h	[32]
TPA-3CPA		25.44	1.193	85.56	26.27	90	The same condition for 800 h	
NiO_x_/TCEP		26.18	1.19	84.7	26.34	~ 97.5	55 °C, N_2_, 1000 h (MPPT)	[134]
MeO-PhPACz	Cs_0.05_FA_0.8_MA_0.15_Pb(I_0.7_5Br_0.25_)_3_	21.14	1.211	84.36	21.60	80	RT^*c*^, N_2_, unencapsulated, 1000 h (MPPT)	[33]
2PhPA-CzH		21.27	1.238	86.70	22.83	95	The same condition, 1000 h (MPPT)	
Ph-4PACz	Cs_0.05_(FA_0.98_MA_0.02_)_0.95_Pb(I_0.98_Br_0.02_)_3_	25.00	1.20	83.6	25.01	> 80	65 ± 5 °C, N_2_, 1000 h (MPPT)	[40]
BCzPA		25.65	1.158	82.10	24.43	76	65 °C, N_2_, unencapsulated, 500 h (MPPT)	[37]
Th-BCzPA		25.83	1.170	84.28	25.48	85		
Ph-BCzPA		25.78	1.188	85.99	26.33	93		
4PACz	Cs_0.05_MA_0.10_FA_0.85_PbI_3_	25.27	1.161	81.29	23.85	90.1	RT, N_2_, unencapsulated, 1000 h (MPPT)	[69]
Me-TPCP		25.67	1.185	84.21	25.62	70.3		
2PACz	Cs_0.05_FA_0.93_MA_0.02_Pb(I_0.95_Br_0.05_)_3_	23.86	1.106	83.32	22.02	< 70	100 ± 2 °C, N_2_, stored for 800 h	[90]
2,7CZ-F		24.81	1.118	83.39	23.13	< 70	The same condition, stored for 800 h	
2,7CZ-FV		25.48	1.131	83.86	24.17	90	The same condition, stored for 1000 h	
PTAA	Cs_0.2_FA_0.8_Pb(I_0.6_Br_0.4_)_3_	17.28	1.191	81.69	16.82	< 60	N_2_, unencapsulated, 2 h (MPPT)	[139]
BCB10Br-C4PA		17.54	1.286	82.58	17.29	90	The same condition, 250 h (MPPT)	
MPA2FPh-BT-BA		17.93	1.31	82.31	19.33	–	–	[57]
DCB-C4POH	Cs_0.2_FA_0.8_Pb(I_0.6_Br_0.4_)_3_	17.64	1.31	82.31	19.08	–	–	[102]
DCB-Br-1		17.70	1.33	82.81	19.54	–	–	
DCB-Br-2		18.21	1.37	83.53	20.76	–	–	
4PACz	(Cs_0.03_FA_0.82_MA_0.15_)Pb(I_3-x_Cl_x_)	25.92	1.135	81.99	24.12	79	65 °C, N_2_, 1000 h (MPPT)	[79]
DMICPA		26.06	1.176	85.65	26.27	93		
DBPP	FA_0.3_MA_0.7_PbI_3_	25.1	1.15	79.1	22.8	70	65 °C, RH 40% in air, encapsulated, 1000 h (MPPT)	[38]
Poly-DBPP		25.7	1.18	82.9	25.1	92	The same condition, 1600 h (MPPT)	
PhPACz		24.5	1.14	77.4	21.6	11.7	40 °C, RH 30–40% in air, 800 h(MPPT)	[36]
Poly-PhPACz		1.18	26.2	84.3	26.1	92	The same condition, 1800 h (MPPT)	
DCPA		25.4	1.13	79.4	22.0	30	85 °C, RH 50 ± 10% in air, encapsulated, 400 h (MPPT)	[35]
Poly-DCPA		25.0	1.17	83.6	24.9	94	The same condition, 900 h (MPPT)	

MPPT^a^: maximum power point tracking, RH^b^: relative humidity, RT^c^: room temperature

However, SAMs suffer from stability issues under operational condition (e.g., UV, humidity and temperature fluctuations) due to their simple monolayer structure, fragile chemical bonds, and small molecular weight. While the monolayer nature of SAMs undoubtedly confers advantages in achieving near-lossless interfacial contact, it also renders devices highly susceptible devices to minor perturbations [16, 17]. These undesirable changes can contribute to critical deterioration at the perovskite interface by causing direct contact between the constituents, thereby deteriorating current leakage, and strengthening non-radiative recombination processes and other harmful effects. Studies has demonstrated that interface modification, NiO_x_/SAMs bilayer, and co-SAMs strategy can address partial issues mentioned above [18, 23, 87, 132, 154–159]. Despite the significant progress in the recent years, several challenges regarding SAM and co-SAM still need to be tackled. Future studies may be directed toward, but not limited to the following directions:

1. Advanced Characterization: How to obtain monolayer with high ordered orientation and compact packing is still challenging. The fundamental properties of co-SAMs remain unclear and merit in-depth investigation. For example, it is still debated how the anchoring groups are exposed—on the surface or become wrapped internally? Advanced in situ characterization techniques, including in situ AFM [166], TEM, and STM, are expected to provide critical insights into the atomic-scale structure and molecular organization of SAM and co-SAM layers. In addition, SERS can be combined with STM probe microscopy to realize TERS to achieve Raman signal mapping [96].
2. Molecule Design: The exploration of SAMs with precisely tailored molecular sizes, configurations, and functional groups continues to represent a vital and highly promising research direction. Nowadays, universal monodentate anchoring group –PO(OH)_2_ shows strong corrosivity and insecure connection with the substrate. Developing weakly acidic and multidentate anchoring groups remains highly valuable. For terminal groups, it strongly influences surface wettability, work function, and interfacial carrier transfer. The benzene ring, leveraging intermolecular π-π interactions, can modulate the stacking behavior of SAMs, promote densely packed organization, and favor face-on molecular orientation. Designing multifunctional terminal groups is still challenging due to multifunctionality dilemma, space steric hindrance, complex orientation control, and competitive interface reaction. For linker groups, employing short conjugated chains (such as benzene rings, thiophenes) to balance conductivity and orientation control is currently the best choice due to insulation of alkyl chains. However, rigid π-conjugate linker groups present challenges in molecular orientation and aggregation. Besides, rigid π-conjugate hinders the effective contact between the anchoring group and the substrates. During molecular design, solubility and balance of driving forces should be considered. The molecules must exhibit appropriate solubility in the assembly solvent to enable uniform adsorption. The adsorption of anchoring groups onto the substrate, intermolecular interactions (van der Waals forces, π-π stacking), and interactions between terminal groups should act in synergy to drive the formation of an ordered, low-defect monolayer, rather than disordered multilayers or island-like structures.
3. Scalable Fabrication: Although a lot of fabrication methods such as spray coating, evaporation, slot die, and blade coating have been reported to process SAMs with large area. The large-scale fabrication of SAM-based IPSCs remains challenging, mainly due to significant efficiency losses. This issue highlights considerable potential for optimization, not only in terms of perovskite composition and the deposition methods of functional layers but also in the molecular design of SAMs—particularly to reduce aggregation, which critically influence device performance. Additionally, the stability of large-area IPSCs represents another crucial consideration. As previously noted, anchoring groups with mild acidity show great promise for use in large-area IPSC fabrication. In comparison with devices employing only a SAM, the NiO_x_/SAM bilayer structure exhibits superior performance in forming conformal, uniform, and stable hole-selective contacts, which is advantageous for the scalable manufacturing of efficient and stable IPSCs. Poly-SAMs, processing multianchoring sites and interchain entanglement, can perfectly cover the substrate and hold great potential for large-scale fabrication. Covalently linked polymer networks and strong interchain forces (such as cross-linking) endow the poly-SAMs with high toughness and adhesion. Unlike small molecules, the orientation of the terminal groups and the arrangement of the chain segments in polymer chains are more difficult to control uniformly, especially for flexible chains or complex linker copolymer. Nevertheless, further improvements are still required in both the deposition processes and the molecular design of SAMs.

There is still a long way to go for SAMs from the laboratory to practical applications. In the future, it is necessary to find new deposition methods to improve the uniformity and stability of SAMs in IPSCs and increase the PCE of devices. By designing new SAMs and expanding their applications in different devices, SAMs will play an increasingly important role in the future.
